# Gap junction protein Connexin-43 is a direct transcriptional regulator of N-cadherin in vivo

**DOI:** 10.1038/s41467-018-06368-x

**Published:** 2018-09-21

**Authors:** Maria Kotini, Elias H. Barriga, Jonathan Leslie, Marc Gentzel, Verena Rauschenberger, Alexandra Schambony, Roberto Mayor

**Affiliations:** 1 Department of Cell and Developmental Biology, University College London, Gower Street, London, WC1E 6BT UK; 2 London Centre for Nanotechnology, University College London, London, WC1H 0AH UK; 3 Max Planck Institut for Molecular Cell Biology and Genetics, Pfotenhauerstr. 108, 01307 Dresden, Germany; 4 Biology Department, Developmental Biology, Friedrich-Alexander University Erlangen-Nuremberg, Erlangen, 91058 Germany; 5 Present Address: Center for Molecular and Cellular Bioengineering, Facility Molecular Analysis/Mass Spectrometry, TU Dresden, Tatzberg 47/49, 01307 Dresden, Germany

## Abstract

Connexins are the primary components of gap junctions, providing direct links between cells under many physiological processes. Here, we demonstrate that in addition to this canonical role, Connexins act as transcriptional regulators. We show that Connexin 43 (Cx43) controls neural crest cell migration in vivo by directly regulating N-cadherin transcription. This activity requires interaction between Cx43 carboxy tail and the basic transcription factor-3, which drives the translocation of Cx43 tail to the nucleus. Once in the nucleus they form a complex with PolII which directly binds to the N-cadherin promoter. We found that this mechanism is conserved between amphibian and mammalian cells. Given the strong evolutionary conservation of connexins across vertebrates, this may reflect a common mechanism of gene regulation by a protein whose function was previously ascribed only to gap junctional communication.

## Abstract (web summary)

Connexins are components of gap junctions that link cells and allow intercellular communication. Here, the authors show that the Connexin 43 carboxy tail interacts with basic transcription factor-3, leading to nuclear translocation and direct regulation of N-cadherin expression and neural crest migration.

## Introduction

Gap junctions are transmembrane complexes of connexin proteins that allow intercellular communication and the transfer of ions and small signaling molecules between adjacent cells^1^. In addition to their channel functions at the plasma membrane, connexins can produce small fragments or isoforms that are present in different cellular compartments including the nucleus^2^ and thus may function in alternative processes, such as gene expression^3,4^.

The mutual regulation in the assembly of gap and adherens junctions^5,6^ suggests a possible coordination in the expression of their constituent proteins. Collective cell migration, which is fundamental for morphogenesis and cancer invasion^7^, depends on both gap and adherens junctions^8^. In mice, the gap junction protein Connexin 43 (Cx43) is essential for the formation of heart structures like the conotruncus. This role is attributed to the function of Cx43 in cardiac neural crest cells, which migrate to the target tissue and contribute to heart development^9,10^. In many systems, embryonic neural crest cells can undergo collective cell migration^7,11^ and require a tight regulation of the expression of the adherens junction protein N-cadherin^12,13^. Both N-cadherin and Cx43 modulate cell migration^14,15^ and their interaction has been furthered explored in mesenchymal cells, where Cx43 was shown to modify the levels of N-cadherin at the cell membrane^16^. However, the mechanism driving this regulation remains unknown.

Here, we ask whether Cx43, one of the most widely studied gap junction proteins, regulates N-cadherin expression during collective cell migration and investigate the molecular nature of such regulation. We show that Cx43, a molecule primarily known for its membrane-linked activities, uses its tail isoform to control morphogenetic movements via transcriptional regulation of N-cadherin. This nuclear activity is independent of its function as channel in the cell membrane. Moreover, we identify its mechanism of action, showing that Cx43 regulation of N-cadherin is due to a direct interaction with the basic transcription factor 3 (BTF3). BTF3 is able to form a stable complex with polymerase II and is part of the transcription initiation complex^17,18^. In more recent studies, BTF3 upregulation has been correlated with tumor prognosis^19,20^ and the transcriptional activity of BTF3 has been implicated in proliferation and cancer progression^20,21^. Here, we demonstrate that Cx43-tail, BTF3 and Pol II altogether form a complex that directly binds to the n-cad promoter to modulate N-cadherin transcription. Furthermore, we show that this unexpected activity of Cx43 as a regulator of N-cadherin is conserved between amphibian and mammalian cells.

## Results

### Cx43 promotes neural crest migration via N-cadherin regulation

**Fig. 1:**
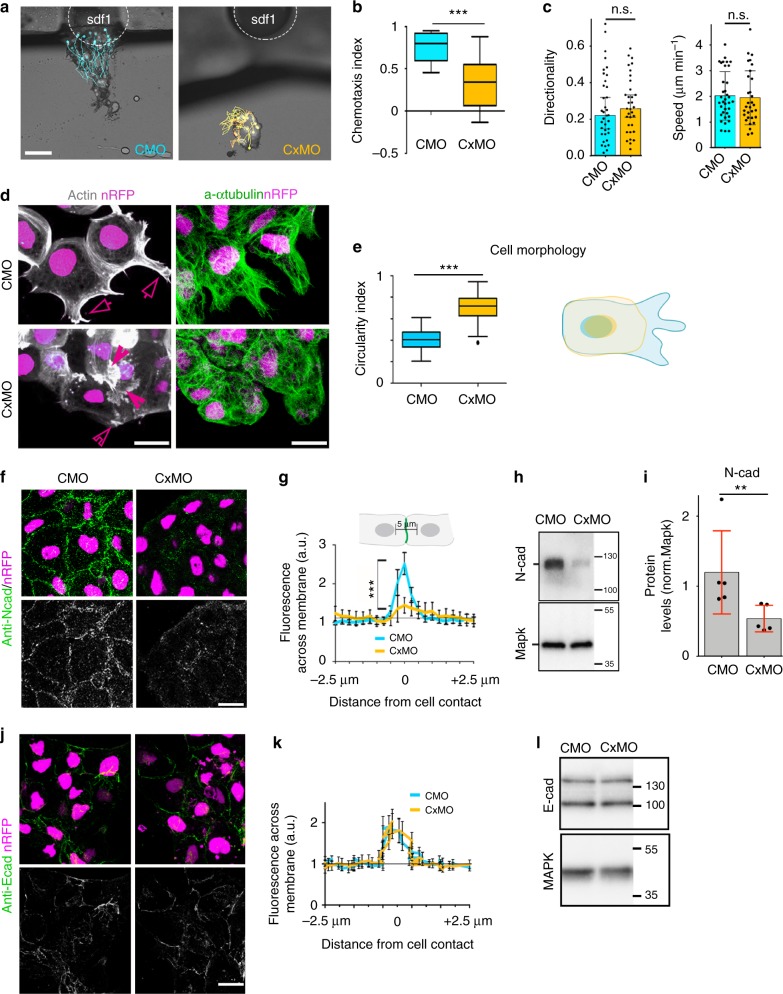
Cx43 controls NC migration via N-cadherin regulation. **a** Neural crest chemotaxis towards SDF-1. Scale bar = 100 μm. **b** Chemotaxis index (*n*_CMO_: 40, *n*_CxMO_:45 explants; *N* = 3). **c** Directionality and velocity of stage 23 (st23) single neural crest cells. Neural crests were dissected and dissociated as described in Methods, followed by time-lapse imaging and tracking of individual cells (*n*_CMO_: 110, *n*_CxMO_: 115 cells, *N* = 4), related to Supplementary Movie 1. **d** Neural crest cell morphology as visualized by life-actin-GFP (arrows mark normal and arrowheads irregular protrusions) and by immunostaining for α-tubulin. **e** Cell morphology quantification (*n*_CMO_ = 58, *n*_CxMO_ = 39, *N* = 3) and diagram: control cell is shown in blue and Cx43 morphant in yellow. **f** N-cadherin (NCad) immunostaining of st23 neural crest. **g** Quantification of N-cad across the cell contact of st23 neural crest. N-cad levels, 0 represents contact point (*n*_CTLMO_ = 25 and *n*_Cx43MO_ = 45 explants, *N* = 3). **h** Western blot (WB) of st23 embryo lysates for N-cad and Mapk. **i** N-cad levels normalized to MAPK (*n*_CMO_: 156, *n*_CxMO_: 128 embryos, *N* = 5). **j** E-cadherin (ECad) immunostaining of st23 neural crest. **k** Quantification of E-cad across the cell contact of st23 neural crest cells. E-cad levels, 0 represents contact point (*n*: 13 explants, *N* = 3). Scale bars in **d**, **f**, and **j** = 20 μm. **l** WB of st23 embryo lysates for E-cad and Mapk. Box plots in **b** and **e** show the median, box edges represent the 25th and 75th percentiles, and whiskers show spread of data including outliers (Mann Whitney test p^***^ < 0.001) and histograms in **c**, **i** mean ± SE (two-tailed *t* test p^**^ < 0.01). Lines in **g** and **k** show mean and error bars represent SD (two-tailed *t* test p^***^ < 0.001). *N* number of independent experiments; *n* sample size. Spread of data in bar charts is shown as overlying dots. n.s. nonsignificant

**Fig. 2:**
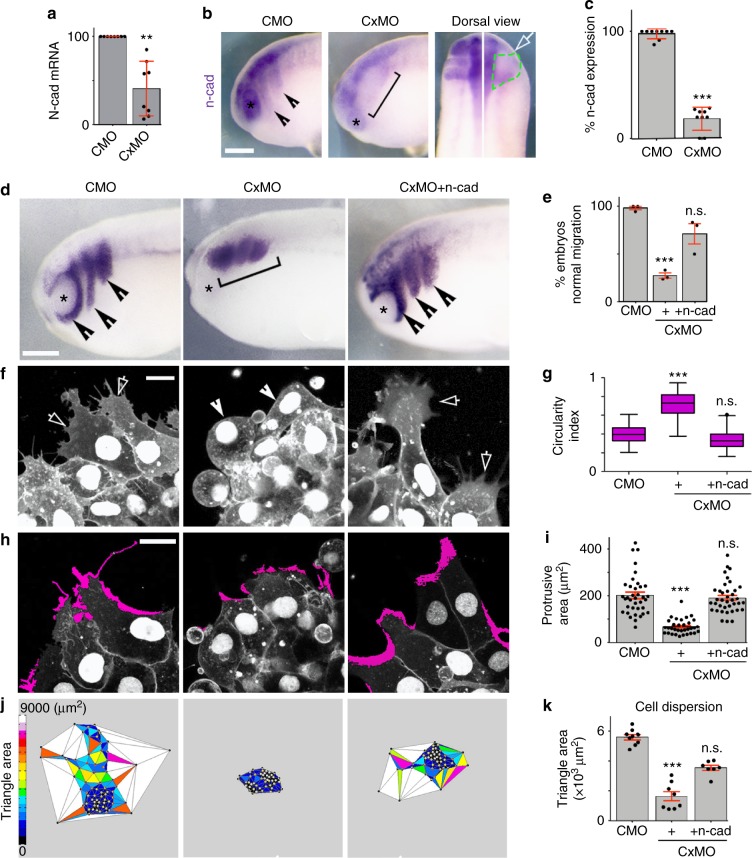
Cx43 mediates N-cadherin mRNA expression. **a** qPCR of *n-cad* (*n*_CMO_ = 68, *n*_CxMO_ = 58 embryos, *N* = 8). **b** Lateral and dorsal views of st23 embryo, analyzed by in situ hybridization ISH against *n-cad*, arrowheads indicate normal and bracket decreased expression, white arrow injected side and green segmented line the NC. **c** % of *n-cad* expressing embryos shown in **b** (*n*_CMO_ = 178, *n*_CxMO_ = 187 embryos, *N* = 9). **d** St23 embryo showing neural crest migration by ISH for *twist*; normal (arrow) and impaired (brackets) migration and **e** % of st23 embryos with normal neural crest migration (*n*_CMO_ = 89, *n*_CxMO_ = 105, *n*_CxMO-ncad_ = 124 embryos, *N* = 3). Scales bar in **b** and **d** = 50 μm. **f** St23 neural crest cells expressing nRFP + mRFP; normal (arrows) and short protrusions (arrowheads) and **g** cell morphology quantification (*n*_CMO_ = 86, *n*_CxMO_ = 87, *n*_CxMO-ncad_ = 98 cells, *N* = 3); Scales bar in **f** and **h** = 15 μm. **h** St23 neural crest cells expressing nRFP + mRFP; in magenta lamellipodia extensions and **i** quantification of protrusion area, (*n*_CMO_ = 37, *n*_Cx43MO_ = 33, *n*_CxMO-ncad_ = 38 cells, *N* = 3). **j** Cell dispersion analysis after 6 h of culture using Delauney triangulation and **k** its quantification (*n* = 10 NC explants, *N* = 3). Histograms in **a**, **c**, **e**, **i**, and **k** represent mean ± SE (two-tailed *t* test p^**^ < 0.01, p^***^ < 0.001). In **g** box plots show the median, box edges represent the 25th and 75th percentiles, and whiskers show spread of data including outliers (Mann Whitney test p^***^ < 0.001). *N* number of independent experiments; *n* sample size. Spread of data in bar charts is shown as overlying dots. n.s. nonsignificant

To examine the role of Cx43 in neural crest development, we used antisense morpholino knock-down (Cx43MO). Depletion of Cx43 impaired collective chemotaxis of *Xenopus* cephalic neural crest (Fig. 1a, b; Supplementary Movie 1), without affecting single cell motility (Fig. 1c; Supplementary Movie 2). On the cellular level, we found that Cx43 is essential for cell morphology and polarization (Fig. 1d, e). We next asked whether downregulation of Cx43 affects expression of N-cadherin, which induces cell polarity and is required for neural crest migration^11–13^. Cx43MO led to a reduction in N-cadherin protein (Fig. 1f–i), whereas the levels of other junctional proteins such as E-cadherin were unaltered (Fig. 1j–l). Analysis by QPCR and in situ hybridization showed that Cx43MO decreased *n-cadherin* at the mRNA level (Fig. 2a–c), The effects of Cx43MO on neural crest migration (Fig. 2d, e), cell polarity (Fig. 2f, g), protrusions (Fig. 2h, i), and cell dispersion (Fig. 2j, k) were rescued by co-expression of *n-cadherin* mRNA, showing N-cadherin as the main Cx43 target in this process. Together these results show that Cx43 promotes neural crest migration by controlling N-cadherin levels.

**Fig. 3:**
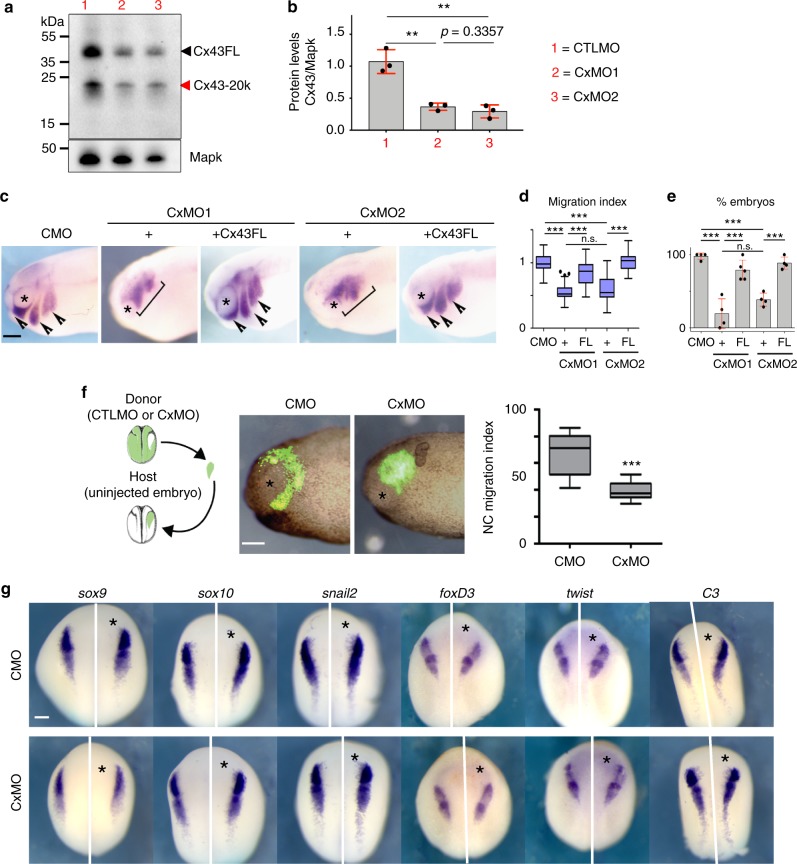
Specificity of Cx43 MOs in neural crest cells. **a**–**c** Effect of two different antisense morpholinos against Cx43 (see Methods). **a** Western blot for Cx43 and α-tubulin, from st23 embryo lysates. **b** Cx43 levels were normalized to α-tubulin and plotted (*N* = 3). **c** Analysis of neural crest migration by ISH for *twist* in st24 embryos, arrows show normal and brackets impaired migration. Co-injection of either of the MOs with human Cx43FL mRNA, which does not bind the MOs, but is highly conserved with *Xenopus* Cx43, showed a rescue in neural crest migration confirming the specificity of these treatments. **d** Neural crest migration index for each treatment. The length of each cephalic neural crest stream was measured in the injected (experimental) and uninjected (control) of the same embryo, and the migration index was defined as the ratio between experimental and control stream length. **e** % of embryos with normal neural crest migration in each treatment (*n*_CMO_: 89, *n*_CxMO1_: 78, *n*_CxMO1+FL:_ 85, *n*_CxMO2_: 74, *n*_CxMO2+FL_: 95, *N* = 4). Note that there is no significant difference between the phenotypes produced by the two morpholinos (n.s. in **d** and **e**). **f** CMO- or CxMO-NC transplanted into uninjected host embryos showing tissue-autonomous function. Migration index of st24 transplanted neural crest (*n*_CMO_ = 35, *n*_CxMO_ = 37, *N* = 3). **g** St 16 embryos were analyzed by ISH for the early expression of neural crest markers *sox9*, *sox10*, *snail2*, *foxD3, twist* or *C3*, asterisk indicates the injected side (*n*_CMO_ = 50; *n*_CxMO_ = 50, *N* = 3). Scale bar in **c**, **f**, and **g** = 50 μm. Histograms in **b** and **e** represent mean ± SE (one-way ANOVA *p* < 0.001, two-tailed *t* test p^*^ < 0.05, p^**^ < 0.01, p^***^ < 0.001). In **d** and **f** box plots show the median, box edges represent the 25th and 75th percentiles, and whiskers show spread of data including outliers (Mann Whitney test p^***^ < 0.001). *N* number of independent experiments; *n* sample size. Spread of data in bar charts is shown as overlying dots. n.s. nonsignificant

Several experiments were performed to control for the specificity of our antisense morpholino treatments. First, 8–24 ng of two different morpholinos were used and because they generated identical efficiency and phenotypes (Fig. 3c), we show the results of only one of them, hereafter referred as Cx43MO. Second, efficiency of the morpholinos was determined by western blot against Cx43 (Fig. 3a, b), whereas specificity was demonstrated by rescue with Cx43 mRNA that does not bind the morpholinos (Fig. 3c–e). In addition, we showed by grafting experiments that Cx43 MO works in a cell autonomous manner (Fig. 3f) and that specifically affect neural crest migration without interfering with neural crest induction, as none of the early neural crest markers are affected by Cx43MO (Fig. 3g). These experiments show that Cx43MO is a specific inhibitor of Cx43 during neural crest migration.

### Cx43 20k isoform controls cell migration via N-cadherin

**Fig. 4:**
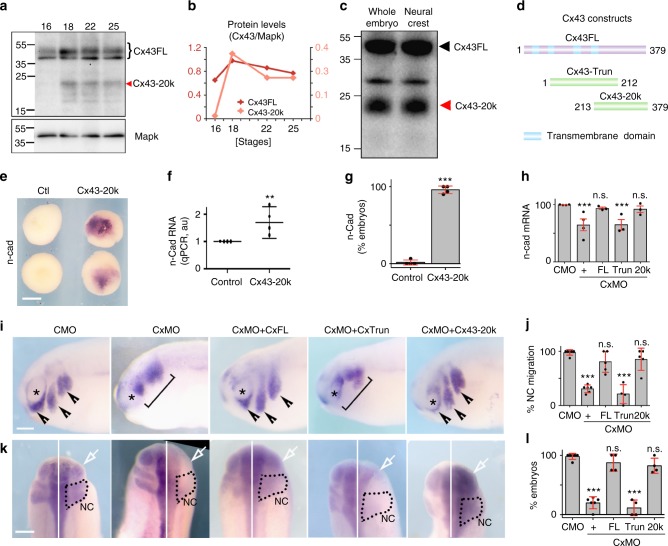
Cx43 20 kDa isoform promotes N-cadherin expression. **a** WB showing Cx43 temporal expression. **b** Cx43 expression levels normalized to MAPK. Levels for Cx43 full length (Cx43FL) and Cx43-20k are plotted (*n* = 82 embryos, *N* = 3). **c** Western blot against Cx43 using extracts from whole embryos (st21) and dissected neural crest (st21) (*n*_whole embryo_ = 20; *n*_neural crest_ = 50 neural crests, *N* = 3). **d** Diagram of Cx43 constructs used. **e** Blastula embryos analyzed by ISH for *n-cad* after the indicated treatments. Scale bar = 70 μm. **f** qPCR for *n-cad* from blastula control embryos versus embryos expressing Cx53Tail (*n*_Control_: 89, *n*_Cx43-20k_: 95 embryos, *N* = 4) and **g** % of blastula embryos expressing N-cadherin mRNA analyzed by ISH (*n*_Control_ = 62, *n*_Cx43-20k_ = 85 embryos, *N* = 4). **h** qPCR for *n-cad* of st24 embryos (*n*_from-left-to-rigth_ = 120, 118, 92, 86, 78, 92 embryos, *N* = 4). **i** St24 embryos showing neural crest migration by ISH of *twist*; arrowheads: normal migration, brackets: impaired migration, asterisk: eye. **j** % of embryos with normal neural crest migration as analyzed by *twist* ISH of st24 embryos (*n*_CMO_ = 115 and *N* = 6, *n*_CxMO_ = 116 and *N* = 6, *n*_CxMO+FL_ = 124 and *N* = 5, *n*_CxMO+Trun_ = 145 and *N* = 4, *n*_CxMO+Cx43-20k_ = 156 and *N* = 5). **k** Dorsal view of St24 embryos analyzed for ISH of *n-cad*; arrows indicate injected side and black dotted area the neural crest. **l** % *n-cad* expressing embryos st24 (*n*_CMO_ = 42 and *N* = 6, *n*_CxMO_ = 42 and *N* = 6, *n*_CxMO+FL_ = 13 and *N* = 4, *n*_CxMO+Trun_ = 15 and *N* = 4, *n*_CxMO+cx43-20k_ = 19 and *N* = 4). Scale bars in **i** and **k** = 60 μm. Histograms in **g**, **h**, **j**, and **l** represent mean ± SE (one-way ANOVA *p* < 0.001; two-tailed *t* test p** < 0.01, p* < 0.05) n.s. nonsignificant. Dots in **f** show the spread of data and lines represent median ± interquartile (Mann Whitney test p** < 0.01). *N* number of independent experiments; *n* sample size. Spread of data in bar charts is shown as overlying dots

It has been shown in mammalian cells that Cx43 exists in two forms: the full-length molecule (Cx43FL), which is a component of gap junctions, and smaller carboxy-terminal fragments, being the most abundant the isoform of 20 kDa (Cx43-20k)^22,23^. These isoforms are present in many cell systems and their function remains unclear. We determined the presence of Cx43 isoforms in *Xenopus* neural crest and examined their temporal expression during development. Cx43FL was expressed at similar levels throughout the developmental stages analyzed, whereas expression of shorter Cx43 isoforms, in particular Cx43-20k, increased just before the onset of neural crest migration (Fig. 4a, b, stage 18 onwards), and we confirmed that these Cx43 isoforms are present in neural crest cells (Fig. 4c).

**Fig. 5:**
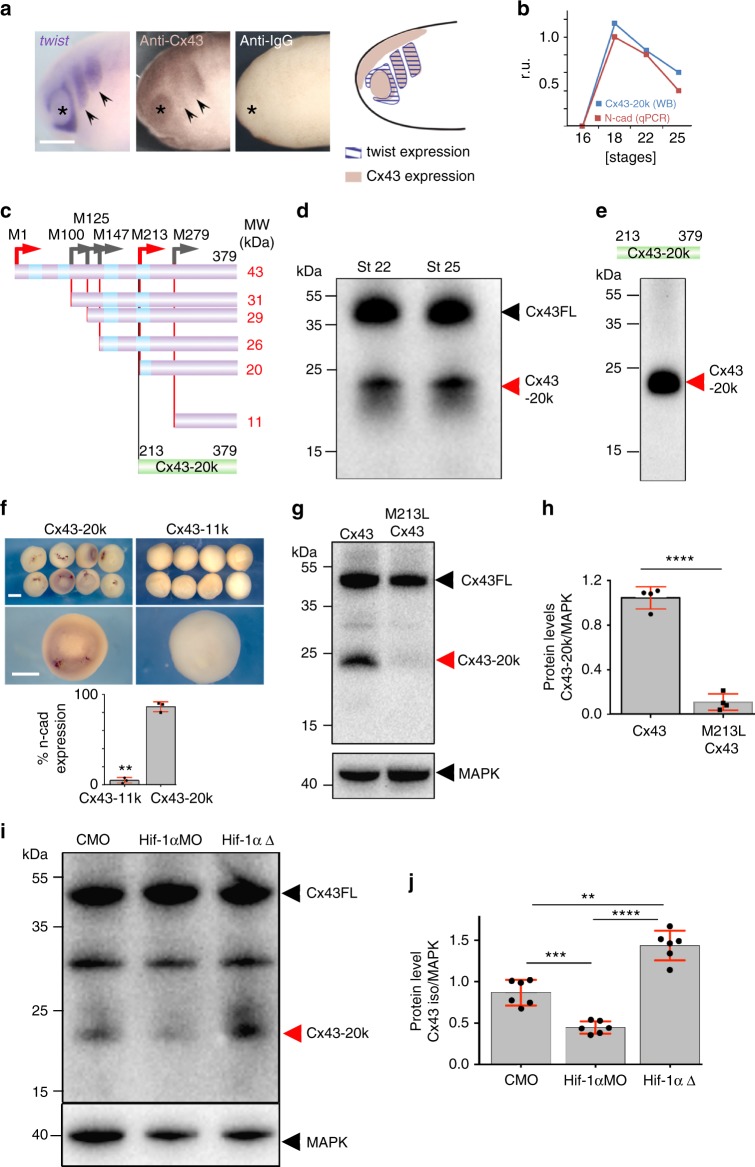
Cx43-20 kDa isoform is generated from Internal Ribosome Entry Site (IRES). **a** *twist* ISH lcompared with Cx43 immunostaining in stage 24 embryos; IgG control antibody; arrow: neural crest; asterisk: eye. Drawing: Cx43 and twist expression summary. Scale bar = 80 μm. **b** Cx43-20k protein levels (analyzed by WB) and N-cadherin mRNA (analyzed by qPCR) in st23 NC cells (*n* = 61 embryos, *N* = 3). **c–h** Cx43 putative IRES and activity of Cx43 isoforms. **c** Predicted methionine-initiated polypeptide. Green: constructs used in **e**. Red arrows: abundant peptides found in *Xenopus*; gray arrows: less abundant or absent peptides in *Xenopus*. **d** WB of embryos at NC migratory stages with antibody against the Cx43 C-terminus. Major bands correspond to Cx43FL and Cx43-20k (arrows). **e** WB of neural crest expressing the Cx43-20k-HA with an antibody against HA. The only band generated corresponds to the M276-initiated peptide (Cx43-20k). **f** Injection of Cx43-20k, but not Cx43-11k, leads to induction of N-cadherin expression in blastula embryos; percentage of embryos displaying N-cadherin expression, (*n*_Cx43-11k_ = 20 embryos *n*_Cx43-20k_ = 20 embryos, *N* = 3). Scale bar = 70 μm. **g** WB against Cx43 from embryos at st21 injected with Cx43-HA (lane 1) and with Cx43 mutated in methionine 213 (M213L Cx43, lane 2). **h** WB’s quantification (*n*_lane1_ = 20 embryos *n*_lane2_ = 20 embryos, *N* = 4). **i** WB of st22 neural crest cells with antibody against the Cx43 C terminus. Lane1: control neural crest; lane 2: neural crest injected with Hif-1α morpholinos; lane 3: neural crest expressing a dominant active form of Hif-1α. **j** Quantification of Cx43 isoforms (*n*_control_ = 50; *n*_Hif-1αMO_ = 50; *n*_Hif-1αΔ_ = 50 neural crests, *N* = 6 independent experiments). Hif-1αMO and Hif-1αΔ were previously validated^25^. In **f**, **h**, and **j** histograms represent mean and bars show s.e.m. (in **j** one-way ANOVA *p* < 0.001, two-tailed *t* test, p^**^ < 0.01, p^***^ < 0.001, p^****^ < 0.0001). *N* number of independent experiments; *n* sample size. Spread of data in bar charts is shown as overlying dots

To test the effects of the Cx43-20k on N-cadherin regulation we selectively over-expressed constructs containing or lacking the 20k fragment (Cx43-20k or Cx43Trun, respectively, Fig. 4d). Expression of Cx43-20k led to upregulation of N-cadherin mRNA in *Xenopus* cells (Fig. 4e–g). Reduction of *n-cadherin* expression and neural crest migration by Cx43MO can be rescued by Cx43FL or Cx43-20k, but not by Cx43Trun (Fig. 4h–l). In addition, Cx43 was coexpressed with N-cadherin in migrating neural crest (Fig. 5a) and the increase in Cx43-20 levels matched those in *n-cadherin* expression (Fig. 5b). These results indicate that the Cx43-20k contains the activity that controls N-cadherin expression and neural crest migration.

Next, we asked how this isoform is generated. Consistently with findings in mammalian cells^22–24^, analysis of the *Xenopus* Cx43 sequence show several putative internal ribosome entry site (IRES) (Fig. 5c); however, the major Cx43 isoform observed correspond to the 20 kDa peptide (red arrow in Fig. 5d). The Cx43-20k fragment contains two putative IRES (M213 and M279) with predicted sizes for the respective polypeptides of 20 and 11 kDa (Fig. 5c). However, expression of the Cx43-20k fragment in *Xenopus* embryos only generates a peptide of 20 kDa (Fig. 5e). Furthermore, expression of the Cx43-20k, but not the Cx43-11k, leads to N-cadherin upregulation (Fig. 5f). To further demonstrate that the Cx43-20k isoform is generated by internal translation we showed that mutation of the M213 blocks the production of Cx43-20k (Fig. 5g, h). Consistently with observations performed in mammalian cells^24^, we observed that the production of Cx43-20k in neural crest cells depends on hypoxia, since the levels of Cx43-20k are reduced or increased during inhibition or activation of the hypoxic factor Hif1α (Fig. 5i, j). Importantly, Hif pathway has been previously shown to be required for neural crest migration^25^. Together, these data show that like in mammalian cell^22–24^, Cx43-20k is produced by the internal translation site M213 generating a 20 kDa peptide that is able to control N-cadherin expression.

We show that the presence of Cx43-20k is conserved across species as it is present in mammalian HeLa cells (Supplementary Fig. 1a) and is able to upregulate N-cadherin levels in HeLa cells (Supplementary Fig. 1b–d) and in amphibian XTC cells (Supplementary Fig. 1e). In addition, inhibition of Cx43 channel activity by two gap junction blockers (F.F.A. and M.F.A.) did not affect N-cadherin expression (Supplementary Fig. 1f–k). Together, these results suggest a role for Cx43-20k in controlling N-cadherin expression and cell migration independent of Cx43 channel activity.

### Cx43-20K isoform is a transcriptional regulator of N-cadherin

**Fig. 6:**
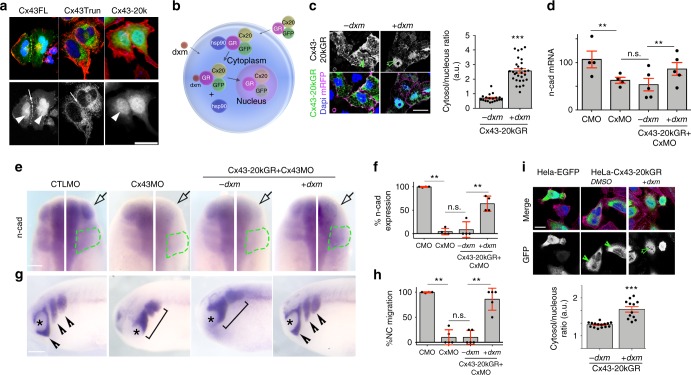
Cx43 carboxy-terminal controls N-cadherin by localizing into the nucleus. **a** Hela cells transfected with GFP-tagged Cx43 forms as indicated (in green), red: phalloidin, blue: DAPI. Scale bar = 25 μm. **b**–**i** Inducible construct to control nuclear localization of Cx43-20k in neural crest and Hela cells. **b** Diagram of Cx43-20k-GR-GFP function. GR: glucocorticoid receptor domain. When Cx43-20kGR is expressed in a cell, it binds to HSP90 which sequestrates it into the cytoplasm; upon addition of the ligand (dex: dexamethasone) Cx43-20kGR is released and it can go into the nucleus. **c** St23 neural crest expressing Cx43-20kGR-GFP with (+) or without (−) dxm. Note that GFP fluorescence is uniformly distributed in -dxm cells, but it becomes localized in the nucleus after dxm treatment. Chart in **c** shows Cx43-20kGR nuclear fluorescence normalized to the cytosolic fluorescence (*n*_−dxm_ = 78, *n*_+dxm_ = 89 cells, *N* = 3). Scale bar = 20 μm. **d** qPCR for n-cad of st20 embryos (*N*_from bars1–4_ = 4, 4, 5, 5). **e** Activation of Cx43-20k nuclear localization by dxm rescues n-cad levels, analyzed by ISH for *twist* in embryos at st24. White arrow shows injected side and green line the neural crest. **f** % of n-cad expressing embryos shown in **e** (*n*_CMO_ = 83, *n*_CxMO_ = 95, *n*_−dxm_ = 67, *n*_+dxm_ = 64, *N* = 4). **g** St24 embryos showing neural crest migration by ISH of *twist*; arrowheads: normal migration, brackets: impaired migration, asterisk: eye. Scale bars in **e**, **i**, and **g** = 50 μm. **h** % of embryos with normal neural crest migration analyzed by ISH for twist; embryos are shown in **g** (*n*_CMO_ = 91, *n*_CxMO_ = 102, *n*_−dxm_ = 87, *n*_+dxm_ = 93, *N* = 5). **i** HeLa cells were transfected with EGFP or with Cx43-20kGR-GFP and treated with DMSO or dxm. Chart in **i** shows Cx43-20kGR-GFP nuclear fluorescence normalized to the cytosolic fluorescence (*n*_−dxm_ = 56, *n*_+dxm_ = 72 cells, *N* = 3); Scale bar = 25 μm. Histograms in **c**, **d**, **f**, **h**, and **i** represent mean ± S.E. (one-way ANOVA *p* < 0.001, two-tailed *t* test p^**^ < 0.01, p^***^ < 0.001). *N* number of independent experiments; *n* sample size. Spread of data in bar charts is shown as overlying dots. n.s. nonsignificant

To understand the underlying cellular mechanism of this transcriptional regulation, we investigated the subcellular localization of Cx43 and Cx43-20k. We observed that Cx43-20k, but not Cx43Trun, localizes in the nucleus of HeLa (Fig. 6a) and neural crest cells (Supplementary Fig. 2a). Furthermore, an antibody that recognizes the carboxy terminal of Cx43 revealed nuclear localization in neural crest (Supplementary Fig. 2b), HeLa cells (Supplementary Fig. 2c) and XTC cells (Supplementary Fig. 2d). This result was confirmed by subcellular fractionation of *Xenopus* embryos, where Cx43-20k was detected in the nuclear fraction (Supplementary Fig. 2e). To investigate if the nuclear localization of Cx43 is required for N-cadherin expression and cell migration, we fused the binding domain of the human glucocorticoid receptor (GR) with GFP-tagged Cx43-20k (Cx43-20kGR-GFP; Fig. 6b). This construct entered the nucleus of neural crest and HeLa cells only in the presence of dexamethasone (dxm)^26^ (Fig. 6c, i). Inhibition of *n-cadherin* expression (Fig. 6d–f) and cell migration (Fig. 6g, h) induced by Cx43MO was rescued in embryos injected with Cx43-20kGR-GFP after dxm treatment (Fig. 6d–h). These results suggest that the nuclear localization of Cx43-20k is required for N-cadherin regulation and that this regulation is conserved across different cell types.

**Fig. 7:**
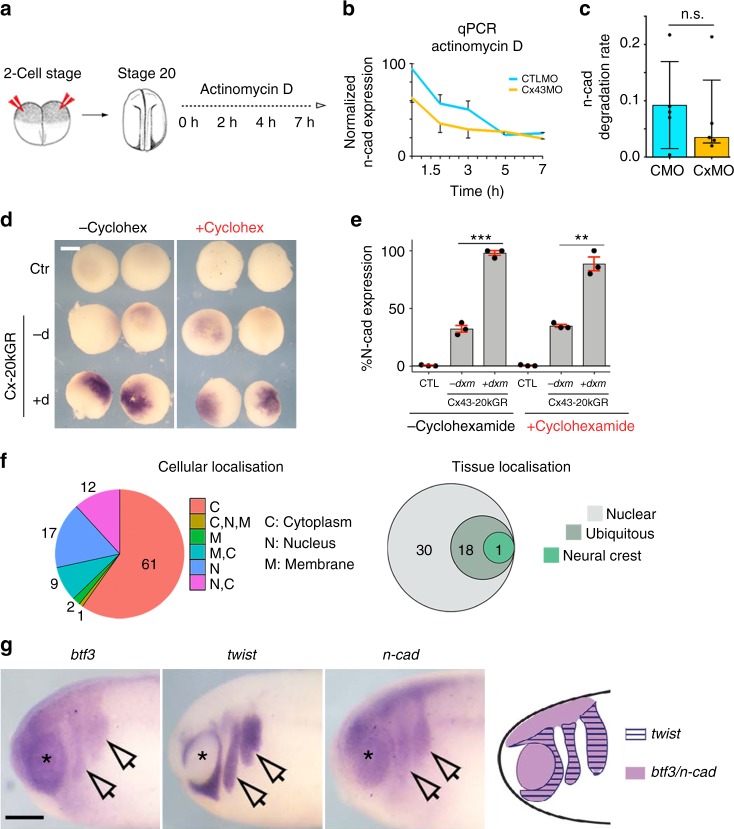
Cx43-20Kd isoform interacts with BTF3 to regulate N-cadherin transcription. **a** Summary of the actinomycin D treatment. CxMO injection in embryos at the two-cell stage, followed by incubation with 4 μg/ml of the transcriptional inhibitor actinomycin D and harvested for qPCR at the indicated times. **b** qPCR of st23 neural crest plotted as relative *n-cad* expression over time. The slope of these curves represents the N-cad mRNA degradation rate on time, which is shown in **c** (*N* = 5). **d** Blastula embryos analyzed by ISH for *n-cad* after the indicated treatments. Scale bar = 40 μm. **e** Quantification of data shown in **e** as % of embryos expressing *n-cad* analyzed by ISH at blastula stage embryos (*n*_CTL_ = 35, *n*_Cx43-20kGR_ = 46, *n*_Cx43-20kGR+dxm_ = 55, *n*_CTL+cyc_ = 65, *n*_Cx43-20kGR+cyc_ = 57, *n*_Cx43-20kGR+dxm+cyc_ = 64, *N* = 3). **f** Summary of mass spectrometry results to identify Cx43-20kD binding partners. Left: Pie chart of Cx43-20 kD interactants. Right: Venn diagram of interactants found in nucleus. The Cx43-20kD binding partner shown in green corresponds to BTF3. **g** In situ hybridization for *btf3*, *twist*, and *n-cad* in st24 embryos, arrows show neural crest and asterisk the eye. Scale bar = 50 μm. Note that *btf3* and *n-cad* are coexpressed in the migrating neural crest (identified by *twist* expression), and in the eye and neural tube. Summary of *btf3*, *twist* and *n-cad* expression profiles. Histograms in **c** and **e** represent mean ± S.E. (in **e** one-way ANOVA *p* < 0.001, two-tailed *t*- test p^**^ < 0.01, p^***^ < 0.001). *N* number of independent experiments; *n* sample size. Spread of data in bar charts is shown as overlying dots. n.s. nonsignificant

We next explored the role of Cx43-20k on N-cadherin regulation. It is possible that Cx43-20k controls *n-cadherin* mRNA levels by regulating transcription or mRNA degradation. We found no difference in the rate of *n-cadherin* mRNA degradation between CTLMO and Cx43MO embryos (Fig. 7a–c), indicating that Cx43 does not regulate mRNA stability. To test whether Cx43-20k controls N-cadherin without the need of translation of other genes, we blocked protein synthesis using the translation inhibitor cycloheximide (CHX). Cx43-20kGR-injected embryos treated with CHX, followed by dxm addition, showed an upregulation of N-cadherin expression, similar to non-CHX treated embryos (Fig. 7d, e), suggesting a direct effect of Cx43-20k on *n-cadherin* transcriptional regulation.

### Cx43-20k isoform interacts with the transcription factor BTF3

**Fig. 8:**
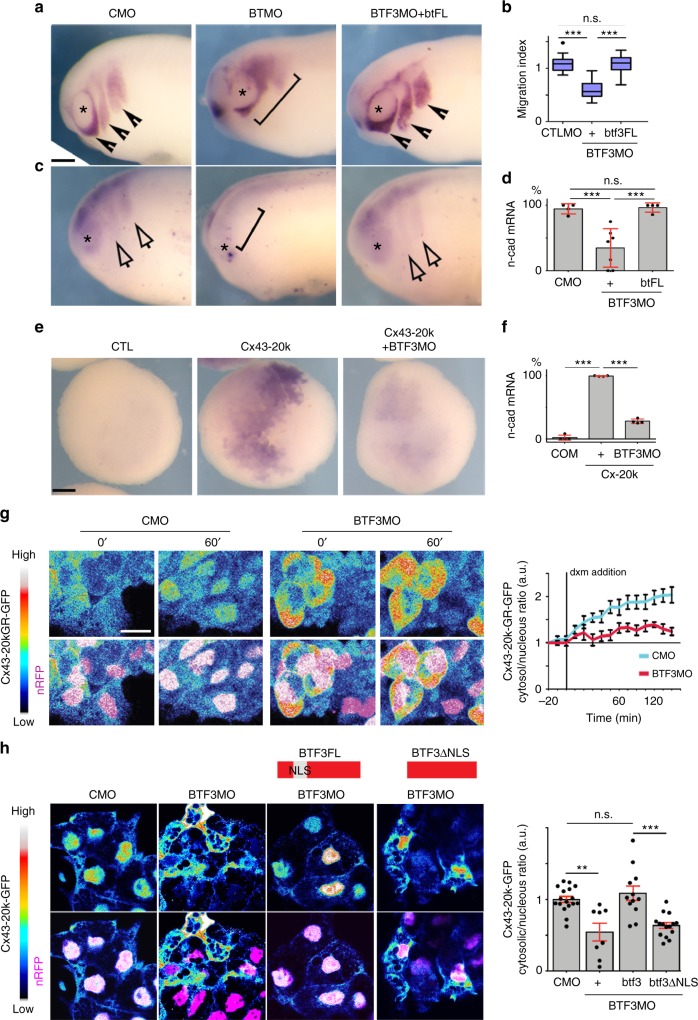
Cx43 carboxy-terminal is recruited to the nucleus by BTF3. **a** St24 embryos showing neural crest migration by ISH of *twist*. Arrowheads show normal and brackets impaired migration. Asterisk indicates the eye. Scale bar = 40 μm. **b** Neural crest migration index for the indicated treatment. (*n*_CMO_ = 89, *n*_BTF3MO_ = 98, *n*_BTF3MO-BTF3FL_ = 105, *N* = 4. **c** Lateral view of embryos at st24 showing *n-cad* expression, analyzed by ISH. Arrows show normal and brackets impaired expression, asterisk indicates the eye. **d** % of n-cad expressing embryos shown in **c** (*n*_CMO_ = 92, *n*_BTF3MO_ = 127, *n*_btFL+BTF3MO_ = 98 embryos, *N* = 4). **e** Analysis of N-cadherin expression in blastulae embryos (st9). Scale bar = 30 μm. **f** *n-cad* expression levels, representative embryos in **e** (*n*_CMO_ = 56, *n*_Cx-20k_ = 112, *n*_Cx-20k-BTF3MO_ = 88 embryos, *N* = 4). **g** Temporal analysis of Cx43-20k-GR nuclear localization in st23 neural crest after addition of dxm (time is shown in minutes after dxm treatment). Cx43-20k-GFP is shown in color-coded intensity and n-RFP is used to visualize the nucleus. Graph shows Cx43-20kGR-GFP nuclear fluorescence normalized to the cytosolic fluorescence (*n*_CMO_ = 25, *n*_BTF3MO_ = 32 explants, *N* = 3). **h** St23 neural crest, showing color-coded intensity of Cx43-20k-GFP compared with nRFP to visualize nuclear localization. In red diagrams BTF3 NLS deletion constructs are shown. Chart of nucleus vs. cytosol ratio of Cx43-20k-GFP (*n*_CMO_ = 25, *n*_BTF3MO_ = 39, *n*_BTF3MO-btf3_ = 58, *n*_BTF3MO-btf3ΔNLS_ = 58, *N* = 3 independent experiments). Scale bars in **g** and **h** = 20 μm. In **b** box plots show the median, box edges represent the 25th and 75th percentiles, and whiskers show spread of data including outliers (Mann Whitney test p^***^ < 0.001). Histograms in **d**, **f**, **h**, and lines in **g** represent mean and error bars S.E. (one-way ANOVA; *p* < 0.001; two-tailed *t* test p^**^ < 0.01, p^***^ < 0.001. *N* number of independent experiments; *n* sample size. Spread of data in bar charts is shown as overlying dots. n.s. nonsignificant

**Fig. 9:**
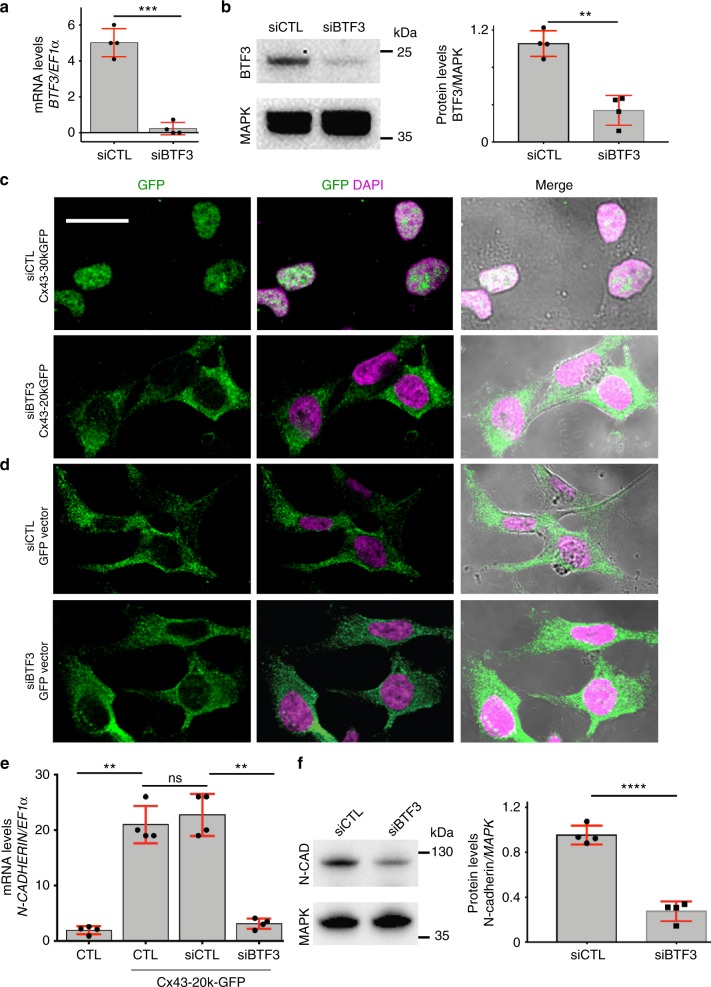
Nuclear localization of Cx43-20k and N-cad expression is BTF3 dependent in HeLa cells. **a**, **b** siRNA efficiency. HeLa cells were transfected with siRNA control (siCTL) and siRNA against BTF3 (siBTF3). siBTF3 leads to an efficient reduction in *Btf3* mRNA as analyzed by qPCR (**a**) (*N* = 4) and BTF3 protein as analyzed by western blot (**b**) (*N* = 4). **c**, **d** Cx43-20k nuclear localization. **c** siBTF3 leads to a strong reduction in Cx43-20k nuclear localization, compared with siCTL (*N* = 3). **d** No difference between siCTL and siBTF3 was observed in cells transfected with a control GFP vector (*N* = 3). Scale bar = 20 μm. **e**, **f** N-cadherin expression. siBTF3 leads to a strong reduction of the *n-cadherin* mRNA (*N* = 4) (**e**) and protein levels (*N* = 4) (**f**) induced by Cx43-20k-GFP, as indicated by qPCR and western blot analyses. Histograms in **a**, **b**, **e**, and **f** represent mean ± S.E. (one-way ANOVA for **e** p^***^ < 0.001, two-tailed *t*- test p^**^ < 0.01, p^***^ < 0.001, p^****^ < 0.0001). *N* number of independent experiments; *n* sample size. Spread of data in bar charts is shown as overlying dots. n.s. nonsignificant

To further explore the mechanisms underlying N-cadherin regulation and translocation of Cx43-20k isoform to the nucleus, we performed mass spectrometry analysis of Hela cells transfected with Cx43-20k construct. Using 1.3-fold change as a minimal cut-off for consideration between immunoprecipitated (IP) and control samples^27^, we found 30 candidates that are nuclear proteins and interact with Cx43-20k (Fig. 7f), reflecting potential binding partners that may be involved in N-cadherin regulation. Examination of the *Xenopus* database (Xenbase) revealed that these 30 candidates are expressed in *Xenopus laevis* but only one is specifically expressed in migrating neural crest cells (Fig. 7f, g): the BTF3^18,28,29^. To test its possible function, we designed a BTF3MO and found that it inhibited neural crest migration and *n-cadherin* expression, similar to Cx43MO, and both phenotypes are rescued by full length *BTF3* mRNA (Fig. 8a–d). BTF3MO decreased endogenous BTF3 levels (Supplementary Fig. 3a), and did not affect neural crest induction (Supplementary Fig. 3b). Co-injection of BTF3MO with Cx43-20k blocked the effects of Cx43-20k alone on *n-cadherin* expression (Fig. 8e, f) and nuclear localization of Cx43-20k (Fig. 8g), indicating that BTF3 is required for Cx43-20k activity. On the other hand, inhibition of Cx43 by Cx43MO did not affect the nuclear localization of BTF3 (Supplementary Fig. 3c), suggesting that BTF3 localizes to the nucleus even in absence of Cx43, whereas Cx43 requires BTF3 to mediate its subcellular localization. These observations are consistent with the presence of a nuclear localization signal (NLS) in BTF3^30^ and its absence in Cx43-20k. Indeed, expression of an NLS-depleted BTF3 construct failed to rescue Cx43-20k nuclear localization (Fig. 8h). Knock-down of BTF3 in mammalian cells (Fig. 9a, b) also impaired Cx43-20k nuclear localization (Fig. 9c, d) and blocked N-cadherin expression induced by Cx43-20k (Fig. 9e, f), arguing for a conserved role of BTF3 and Cx43-20k. Altogether these data show that Cx43-20k is translocated to the nucleus via the NLS domain of BTF3 to control N-cadherin expression in amphibian and mammalian cells.

**Fig. 10:**
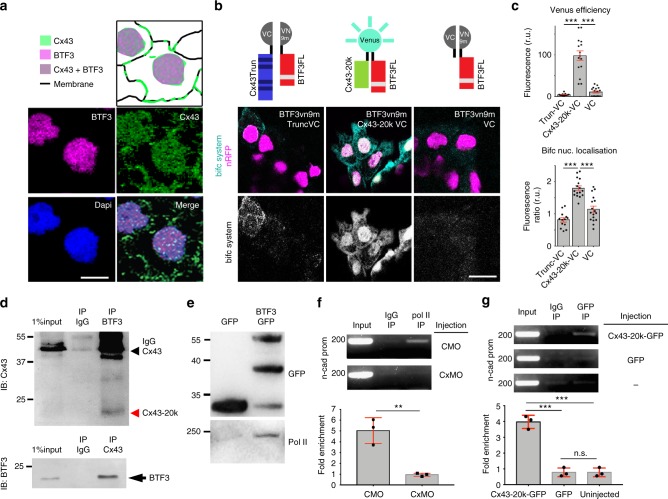
Cx43 carboxy-terminal and BTF3 form a transcriptional complex. **a** Diagram (top) summarizing the localization of BTF3 and Cx43 in the nucleus of st23 neural crest (bottom). Anti-BTF3 (magenta), Anti-Cx43 (green) and DAPI (blue). Scale bar = 15 μm. **b** Diagrams of the BiFC system. BiFC results showing that BTF3 and Cx43-20k interact in the nucleus of st23 neural crest. VENUS components coexpressed on neural crests. Scale bar = 20 μm. **c** Intensity of VENUS showing the efficiency of the BiFC system in the different treatments (top) and nucleus to cytosol ratio (bottom) of BiFC signal (*n*_TruncVC_ = 150, *n*_Cx43-20kVC_ = 147, *n*_VC_ = 98 cells, *N* = 3). **d** Co-immunoprecipitation of BTF3 and Cx43 in HeLa cells (*N* = 4). **e** Co-immunoprecipitation showing biochemical interaction between BTF3 and Polymesare II (*N* = 3). **f**, **g** ChIP experiments for n-cad promoter of st23 neural crest as indicated (**f**: *N* = 3, **g**: *N* = 3). Histograms in **c**, **f**, and **g** represent mean ± SE (one-way ANOVA *p* < 0. 001, two-tailed *t*- test p^**^ < 0.01, p^***^ < 0.001). *N* number of independent experiments; *n* sample size. Spread of data in bar charts is shown as overlying dots. n.s. nonsignificant

As both BTF3 and Cx43 are localized in the nucleus (Fig. 10a) and BTF3 NLS is required for Cx43-20k nuclear translocation, we hypothesized a direct interaction between these proteins. To test for physical interaction between Cx43-20k and BTF3 in the nucleus, we used bimolecular fluorescence complementation system (BiFC), in which two VENUS components (VC and VN9m) form a fluorescent protein when brought into close apposition^31^. To build the BiFC system we fused the VENUS component (VC) to either Cx43-20k or Cx43Trunc, while the other VENUS component (VN9m) was fused to BTF3. Empty VC was used as a control. Cx43-20kVC co-injected with BTf3VN9m gave a positive signal not observed with other combinations (Fig. 10b). Moreover, this signal was higher in the nucleus (Fig. 10c). These data support a direct interaction between Cx43 and BTF3 in the nucleus.

### Cx43-20k isoform and BTF3 binds to *n-cadherin* promoter

To verify the biochemical interaction of Cx43-20k and BTF3, we performed co-immunoprecipitation (IP). We found that endogenous Cx43-20k and BTF3 co-precipitate in Hela cells (Fig. 10d). Our experiments also confirmed that BFT3 interacts with polymerase II (Pol II; Fig. 10e), as previously shown^17^. These results support the notion that three proteins, Cx43Tail, BTF3 and Pol II, interact and may function as a transcriptional complex in the promoter of *n-cadherin*. To test this possibility, we performed chromatin immunoprecipitation (ChIP). We examined whether endogenous Pol II binds to the promoter of *n-cadherin* in the presence or absence of Cx43, by performing PCR against the *n-cadherin* promoter region of CTLMO and Cx43MO ChIP samples (Supplementary Fig. 4). We detected Pol II enriched at the *n-cadherin* promoter in CTLMO ChIP samples, whereas this enrichment was four times lower in ChIP samples collected from Cx43MO-injected embryos (Fig. 10f). These results show that Cx43 is required for Pol II to bind at the *n-cadherin* promoter region. To test whether Cx43-20k is recruited to the *n-cadherin* promoter, we performed ChIP using Cx43-20kGFP or GFP (as a negative control) on embryonic lysates. ChIP-PCR analysis showed that Cx43-20k was enriched by three folds at the *n-cadherin* promoter region (Fig. 10g). Therefore, we propose that Cx43-20k together with BTF3 and Pol II form a complex that controls the transcriptional activity of *n-cadherin* at its proximal promoter.

## Discussion

Although it was formerly speculated that connexin proteins may control gene transcription^2–4^, there was no direct evidence that this occurred and any underlying molecular mechanism remained unknown. Our results are consistent with previous studies showing that connexin fragments are found within the nucleus^4^, and that inhibition of connexins have significant effects on cell differentiation^2–4^. In previous studies, it has been proposed that the generation of these connexin fragments is controlled by the PI3K/AKT/mTOR pathway in mammalian cells^22^, the Mnk1/2 pathway in various cancer cell lines^23^ and hypoxic conditions^24^. It is plausible that similar mechanisms are at play in our system. Indeed, in neural crest, the PI3K/AKT/mTOR pathway is present^32,33^ and could, therefore, be implicated for the generation of connexin fragments. In our study, we show that Cx43 fragments appear at the onset of neural crest migration which coincide in time and space with the activity of Hif-1α (hypoxia- inducible transcription factor-1), which is known to regulate neural crest migration^25^. Indeed, our data show that the generation of Cx43 isoforms is regulated by Hif-1α in embryonic *Xenopus* neural crest, similar to mammalian^24^.

Here, we show that these connexin fragments directly regulate *n-cadherin* expression. In mesenchymal cells it has been shown that Cx43 and N-cadherin regulate reciprocally each other’s levels on the cell surfaces and it has been postulated that N-cadherin and Cx43 share similarities in their regulatory pathways^16^. Such regulation was mainly attributed to their functions at the cell membranes^16,34^. Our results show that Cx43 controls the transcription of N-cadherin, which in turn is required for directional cell migration and cell polarization, a finding that is consistent with known activities of Cx43 in cardiac neural crest in mice^14,15^.

Here, we show that Cx43, independent of its regulation on the cell membrane, via its small isoform that localizes at the nucleus directly affects n-cadherin transcription. It is tempting to speculate that Cx43 may control expression of other genes, in other cell types. Former in vitro studies have proposed an indirect role for Cx43 in gene regulation, such as with members of the CCN family or Smads^31,35–37^. Our data demonstrate that the carboxy-terminal fragment of Cx43 plays a direct role as a transcriptional regulator of gene expression in vivo. Several transcription factors have been shown to regulate *n-cadherin* gene expression such as FoxD3^38^ or factors from Sox family, which bind to the enhancer regions of *n-cadherin* gene^39^. Gene regulatory networks for the neural crest place N-cadherin downstream of these transcription factors^40^, and our results place Cx43 as an additional regulator of *n-cadherin* in the neural crest gene regulatory network.

Here, we show that the transcription factor BTF3 binds to the promoter region of *n-cadherin*^41^ with the help of Cx43 and together positively regulate n-cadherin expression. Thus, BTF3, a transcription factor previously associated with gene expression in cancer systems^20,21^, regulates embryonic gene expression in neural crest, a cell population that has been likened to cancer cells^42^. Cx43 has been postulated to be upregulated in later stages of neural crest development^40^ as in several cancer systems^43,44^, in order to modulate cell migration. Here, we provide evidence that three components associated with embryonic and cancer cell biology are part of a common pathway, an adhesion molecule (N-cadherin), fragments of another adhesion molecule (Cx43) and a transcription factor (BTF3).

In summary, we have identified an original pathway by which an adherens junction component, *n-cadherin*, is regulated by a protein normally associated with a gap junctional communication. This opens the possibility of cross-talk between processes mediating intercellular communication and cell–cell interaction, unveiling a mechanism of cross-regulation between gap and adherens junctions at the transcriptional level. The high conservation of Cx43 across different taxa and the presence of Cx43Tail in various vertebrate systems may reflect an evolutionary conserved mechanism of gene regulation by a protein previously well-known by its gap junction activity.

## Methods

### Embryo manipulation

Adult *X. laevis* were maintained at 17 °C and used according to the regulations of the Biological Service Unit at University College London, complying with the UK Home Office guidelines established in the Animals Act 1986 as described^45^. *X. laevis* embryos were obtained and staged as previously described^46,47^. To specifically target the neural crest, embryos were injected into the animal ventral blastomeres at the eight-cell stage on one side for all experiments, except when embryonic lysates were used. In this case embryos were injected in both animal sides of two-cell stage embryos. Embryo microinjections were performed according to^48^. If required, fluorescein-dextran (FDx; Invitrogen, D1821, 20 ng) or rhodamine-dextran (RDx; Invitrogen, D1824, 20 ng) were used as tracers. Oligomorpholinos against *X. laevis* Cx43 (Cx43MO1; 8–24 ng, 5′-TTCCTAAGGCACTCCAGTCACCCAT-3′, Cx43MO2; 8–24 ng, 5′-AAAAATGGTTTTCTTGTGGGTCGA-3′) and BTF3 (BTF3MO, 40 ng, 5′- AACGGACCGGGTTTAAAGGCTTCCT-3′) were synthesized and provided by Gene Tools LLC. Equimolar concentrations of standard control morpholino (CTLMO: 3′-ATATTTAACATTGACTCCATTCTCC-5′) were used. The amounts of morpholinos correspond to the amounts injected in one side of eight-cell stage embryos, while the other side was used as an internal control. When embryos were used for collection of embryonic lysates, they were injected at two-cell stage in both animal blastomeres and double of the mentioned dosages were used. To test the efficiency of Cx43MOs on the endogenous *cx43* mRNA, we performed western blot analysis. Both Cx43MOs efficiently lowered the protein levels of the endogenous Cx43FL and Cx43iso (Fig. 3a, b). In order to validate BTF3MO efficiency, we tested by immunostaining of neural crest cells the BTF3 protein expression, which showed decreased levels of BTF3 in BTF3MO cells compared to CTLMO (Supplementary Fig. 3a).

In situ hybridization was performed as previously described^49,50^. Briefly, embryos were fixed in MEMPFA, followed by overnight hybridization with digoxigenin-labeled probes, incubated with an AP antibody and AP activity was developed using NBT/BCP substrates. Digoxigenin-labeled RNA probes were prepared for *foxD3*^48^ *snail2*^51^, *twist*^52^, *n-cadherin*^53^, *C3*^53^, *btf3* (Xenbase: XL075i22), and *sox10*^54^, *sox9*^54^. *C3* (1 μg/ml), *snail2* (0.8 μg/ml), *foxD3* (2 μg/ml), *sox10* (1 μg/ml), *sox9* (1 μg/ml), and twist (0.7 μg/ml) were used to assess neural crest induction, *twist* (0.7 μg/ml) for neural crest migration, n-cadherin (1 μg/mL) to assess expression levels and *btf3* (0.7 μg/mL) expression pattern. Whole-mount immunostaining was carried out as previously described^55^ using Cx43 antibody (1:1000, Sigma, C6219). Briefly, embryos were fixed in MEPMFA and incubated overnight with the antibody at the described dilution.

Animal cap explants were dissected from *Xenopus* blastulas (stage 8) using a standard technique^48,56^ and prepared for in situ-hybridization as described above. For animal cap assays, 500 pg mRNA was injected into the animal side of the two blastomeres of two-cell stage embryos. Analysis of ISH was performed on 20–25 embryos per condition for each independent experiment.

### Neural crest manipulation and imaging

Neural crest transplants were carried out as previously reported^57^. Briefly, neural crest taken from stage 18 fluorescently labeled embryos were dissected with eyebrow knives and grafted into unlabeled host embryos. For in vitro experiments, cranial neural crest explants were dissected at stage 18 using a standard technique^58,59^ and plated on fibronectin (Sigma, F1141) coated dishes as previously described^53^. For single cell assays, neural crest cells were briefly dissociated in Ca2+/Mg2+-free Danilchick medium^53^. For each condition, ten embryos transplanted with fluorescent labeled NC were analyzed per experiment.

Immunostaining was performed on neural crest explants as previously described^54^ using anti-N-cadherin (1:50, rat IgG, clone MNCD2, DSHB), anti-E-cadherin (1:250, mouse IgG, clone 5D3, DSHB), anti-BTF3 (1:100, Abcam, ab107213), anti-rabbit IgG Alexa488 (1:500, Invitrogen, A11034) and anti-rat IgG Alexa488 (1:500, LifeTechnologies). When required, 4′,6- diamidino-2-phenylindole (DAPI; 1 μg/mL, Sigma, D9542) and/or phalloidin tetramethylrhodamin-b-isothiocyanate (PhR; 1 μg/mL, Sigma, P1951) were used.

Imaging of fixed embryos was performed using a MZFLIII Leica fluorescence stereomicroscope equipped with a DFC420 Leica camera controlled by Leica IM50 software. Imaging of in vitro neural crest migration was performed using time-lapse cinematography as described previously^53,60^. Briefly, neural crest cells were cultured in plastic or glass petri dishes coated with fibronectin, and time-lapse microcopy was started after one hour of in vitro culture. For cell motility and migration, compound microscopes (either an Eclipse 80i Nikon microscope with a Hamamatsu Digital camera or a DMRXA2 Leica microscope with a Hamamatsu Digital camera controlled by Simple PCI program) equipped with motorized stages and a 10×/0.30NA dry lens were used. NC cultures were prepared as described above. Images were acquired every 5 min for a period of 12 h. For cell morphology imaging, a TCS SP8 microscope with a 63 × /0.90NA water immersion objective lenses and controlled by LAS–AF software was used. Fixed cells were imaged using a 63 × /1.4NA oil immersion objective and a Leica TCS SPE confocal microscope controlled by LAS–AF software.

For construct localization or endogenous IF levels, 10–15 NCCs were analyzed for each condition per experiment.

### Cell migration and cell morphology analysis

Chemotaxis assay was performed following a standard procedure^61^ using heparin acrylic beads (Sigma, H5263) coated with 1 μg/mL purified human stromal cell-derived factor-1 (Sigma, SRP3276). Cell motility and chemotaxis were analyzed using ImageJ (https://imagej.nih.gov) analysis tools, as described previously^53,60^. Briefly, each individual cell was manually tracked using the Manual Tracking plugin of ImageJ, the data were collected and analyzed using the Chemotaxis plugin of ImageJ. Cell morphology was assessed by deploying the circularity index (complete circle = 1) and estimated by ImageJ analysis tools. Cell dispersion was analyzed using Delaunay triangulation algorithm (ImageJ Plugins) and was plotted as average explant triangle area, as described before^54^. Cell protrusive area was analyzed in neural crest cells at the edge of an explant measuring the outgrowing area that derives from two consecutive timeframes with 4 min time interval; these two consecutive frames were subtracted to generate the new area^12^. For analysis of cell chemotaxis and cell dispersion 10–15 explants were analyzed per condition for each independent experiment. For cell motility, cell morphology and cell protrusions 15–25 NCCs were analyzed per condition per experiment.

### Molecular biology, plasmids, and reagents

For cDNA synthesis, total RNA was isolated from 10–15 embryos stages 23–24 or 10–15 animal caps stage 8 from *X. laevis* per condition for each independent experiment and three technical replicas were used within each experiment^25^. Quantitative PCR (qPCR) was performed on an Applied Biosystems ABI 7900HT machine using the Fast SYBR Green Master Mix (Applied Biosystems, 4385612) and the following primers: ef-1 forward 5′- ACCCTCCTCTTGGTCGTTT-3′, ef-1 reverse 5′-TTTGGTTTTCGCTGCTTTCT-3′^15^, ncad forward 5′-CAGGGACCAGTTGAAGCACT-3′, ncad reverse 5′-TGCCGTGGCCTTAAAGTTAT-3′^62^. n-cad mRNA expression was plotted as relative expression normalized against the housekeeping gene ef-1.

Plasmids: Cx43 constructs were synthesized using the *X. laevis* Cx43 sequence from cDNA clone (UniGene ID XL.1109) as template. Full length Cx43 (Cx43FL, aa 1–379), Cx43 carboxy terminally truncated construct (Cx43Trunc, aa 1–212) and Cx43-20k construct (Cx43Tail, aa 213–379) were cloned into 5′BamHI/3′XhoI of pCS2+ or pCS2-EGFP vectors. The pCS2-EGFP vector was kindly provided by Dr. Masa Tada. Inducible construct of Cx43Tail was prepared by fusing the Cx43-20kTail (aa 219–379) to the ligand-binding domain of the human GR (aa 512–777). Cx43-20k was cloned into 5′EcoRI/3′SacI and GR into 5′SacI/3′XhoI of pCS2+ and pCS2-EGFP. BTF3 constructs were synthesized using the *X. laevis* sequence from cDNA clone (UniGene ID XL.3536). BTF3FL (aa 1–162) was cloned into 5′EcoRI/3′XhoI of pCS2+ or pCS2-EGFP. BTF3 deletion construct lacking the NLS region RRKKK (BTF3-dNLS, aa 1–158) was cloned into 5′BamHI/3′ClaI of pCS2+. For the BiFC experiments (Fig. 4b, c), Cx43Tail and Cx43Trunc were cloned into 5′BamHI/3′BamHI of pCS2-VC155. BTF3FL was cloned into 5′BamHI/3′BamHI of pCS2-VN9m. BiFC vectors were kindly provided by Prof James C. Smith. All construct sequences are verified by automated DNA sequencing (Source Biosciences, UK). When required, plasmids were linearized and mRNA transcribed as described before^25^, using sp6MessageMachine (Ambion). The mRNA constructs injected were: membrane GFP (mGFP, 300 pg), membraneRFP (mRFP, 300 pg) nuclearRFP (nRFP, 300 pg), lifeactin-GFP (400 pg), N-cadherin-GFP (500 pg), Cx43FL (500 pg), Cx43Trunc (500 pg), Cx43-20k (500 pg), BTF3FL (500 pg), BTF3-dNLS (500 pg), Cx43-20k-VC (500 pg), Cx43Trunc-VC (500 pg), and BTF3-VN9m (500 pg). The following plasmids were injected as DNA: pcDNA3.2-Cx43-HA (800 pg/embryo,^22^); pcDNA3.2-Cx43-ML-HA (800 pg/embryo;^22^); ΔΜ213 Cx43 (800 pg/embryo,^63^).

All analysis on fluorescent constructs was assessed by normalizing to the background fluorescence and when required, to total cell area fluorescence.

The following reagents were used: flufenamic acid (50 μM for NC explant incubation −100μM for embryo treatment, Sigma, F9005), meclofenamic acid (50 μM for NC explant incubation −100μM for embryo treatment, Sigma, M4531), actinomycin D (20 μM, Sigma, A1410), CHX (10 μM, Sigma, C7698) and ethanol-dissolved dexamethasone (10 μM) was added to the culture medium at stages 14–15 and maintained until neural crest migratory embryonic stages (stage 23). To control the possible leakage of inducible chimeras, a sibling batch of embryos were cultured without dexamethasone and processed for in situ hybridization. For coupling test (testing gap junction channel activity), 10–15 NCCs were analyzed per condition per experiment.

### Immunoprecipitation, fractionation, and western blotting

For each condition 10–15 embryos were used for preparation of embryo lysates per experiment. Whole embryos were prepared for western blot after homogenization in lysis buffer (20 mM Tris, 100 mM NaCl, 0.01% Triton-X, pH 8.0) with added protease inhibitors (Roche, 11836153001) and phosphatase inhibitors (Roche, 04906837001)^54,64^. For western blots, the following antibodies were used: Connexin 43 (Sigma, C6219, 1:1000), N-cadherin (DSHB, clone MNCD2, 1:800), E- cadherin (DSHB, clone 5D3, 1:1000), p42/44 MAPK (Cell Signaling, 9102S, 1:2000), α-tubulin (DSHB, clone 12G10, 1:1000), phospho-histone H3 (Millipore, 06579, 1:2000), GFP (Invitrogen, A11122, 1: 2000), HA-tag (Sigma H6908, 1:2000), rabbit IgG HRP-linked (Amersham ECL, NA934, 1:3000), mouse IgG HRP-linked (Amersham ECL, NA931, 1:3000) and rat IgG HRP-linked (Sigma, A9037, 1:2000). Immunoprecipitation was performed using GFP-Trap^®^ kit (Chromotek); briefly, cells were suspended in lysis buffer and centrifugated at 20,000×*g* for 5 min at 4 °C, the supernatant was recovered and the volume adjusted with dilution buffer. Beads were equilibrated in dilution buffer and mixed with the resuspended cell lysate. The mix was magnetically purified and prepared for western blot. Nuclear fraction isolation of *X. laevis* embryos was performed using differential centrifugation protocols with modifications^64,65^. Briefly, stage 18 *X. laevis* embryo lysates using a 22 G needle and 20 μL homogenization buffer (HB: 250 mM sucrose, 20 mM Hepes, 10 mM KCl, 1 mM EDTA, 1 mM EGTA, 1 mM DTT, phosphatase and protease inhibitors) per embryo. All samples were centrifuged at 250×*g* for 5 min to remove embryo debris. After collecting the supernatant, it was distributed to three different tubes and spun at three different speeds (400×*g*, 600×*g*) for 5 min to isolate the cell nuclei. The postnuclear supernatants were kept for further processing. The nuclear pellets were washed once more in buffer HG and centrifuged at the appropriate speed (400×*g*, 600×*g*). Nuclear pellets were then resuspended in 40 μL 10% glycerol/0.1% SDS/1% Triton-X in HB. Appropriate amount of sample buffer was added to each sample, and samples were processed for acrylamide gel electrophoresis and western blot analysis. In order to avoid loading errors, the same membrane was blotted against the loading control after stripping as described before^54,64^.

Western blot data were analyzed using ImageJ analysis tools. Image intensities were normalized and the ratio of the protein of interest to loading control (i.e., Mapk or α-tubulin) was calculated, average ratios were plotted. Uncropped blots are included as supplementary figures (Supplementary Figs. 5–7).

### Cell cultures

HeLa cells (Leibniz Institute Collections of Microorganisms and Cell Culture, DSMZ, Germany) were cultured in DMEM supplemented with 10% fetal calf serum (Life Technologies, CA, USA) at 37 °C in a humidified atmosphere of 10% CO_2_ and transfected as indicated using Rotifect (Carl Roth, Germany) according to the manufacturer’s instructions.

*Xenopus* embryonic fibroblasts (XTC, a kind gift from Ana Losada) were cultured in 67% DMEM/H2O supplemented with 10% fetal calf serum at 25 °C in a humidified atmosphere of 5% CO_2_ and transfected as indicated using Viafect (Promega, USA). Plasmids for transfection were as indicated and 10–20 Hela or XTC cells were analyzed for each condition per experiment.

For siRNA experiments, a combination of three siRNA targeted against BTF3 were transfected as described above. A standard siRNA (scrambled siRNA from Sigma) was used as a control. Catalog number for these commercial siRNA against BTF3 are: SASI_Hs01_00124567; SASI_Hs02_00308337; SASI_Hs01_00124566. Cells were collected 48 h after transfection and processed for qPCR, western blot, or Immunohistochemestry experiments. As described in the respective sections.

All cell lines were tested for mycoplasma contamination.

### Immunohistochemestry and phalloidin staining

For protein detection, mammalian cells or neural crest explants were fixed in 4% formaldehyde in 0.2% PBS-T (PBS + 0.2% Triton X-100) for 10-min and blocked with 10% NGS for 1 h. Primary antibodies were incubated ON at 4 °C in 10% NGS. The following antibodies were used: 1/100 anti-BTF3 (Abcam, ab107213), 2.5 µg/ml of anti-N-cadherin (MNCD2 Developmental Studies Hybridoma Bank), and 1:100 anti-Cx43 (Sigma, C6219) in 10% NGS and incubated ON at 4 °C. Explants were washed 3 times with PBS + 0.2% Tween-20 and incubated ON at 4 °C with secondary antibody, diluted at 1:350 in 10% NGS. DAPI was diluted at 1:1000 and mixed with the secondary antibodies.

### Mass spectrometry

For co-immunoprecipitation of the FLAG-tagged Cx43Tail for mass spectrometric analysis cells were lysed and lysates were incubated overnight with anti-HA and equilibrated high affinity beads at 4 °C, the immunoprecipitates were then washed and eluted^27^. Subsequently of acid elution with 0.1 M glycine pH 2.5, the pH of the eluates was adjusted to pH 8.0 with 0.5 M Tris. For protein digestion samples were incubated overnight with 2 µg trypsin (Trypsin Gold, Promega, USA) followed by addition of 0.1 µg Lys-C (Roche, Germany) and incubation for additional 6 h. The peptides were desalted on C-18 reversed phase stage tips (Nest Group, USA), dried in vacuum and stored at −20 °C until analysis. Dried peptides mixtures were recovered in 3 µl 30% formic acid and diluted with water to 23 µl. Five µl of the digest were injected on a Nano-LC system (Eksigent425 2D Nano-LC; Sciex, USA) and peptides were separated by reversed phase chromatography on C-18 columns prepared in-house (Reprosil-Pur C18-AQ, 1.9 µm, Dr. Maisch, Germany, PicoFrit Columns, 75 µm i.d., New Obejctive, USA) in a linear gradient of 100% eluent A (0.1% formic acid) to 55% eluent B (60% actenitrile, 0.1% formic acid) in 120 min. The LC-system was setup as vented column and sample was load and desalted at a flow rate of 400 nl/min and separation was carried out at a flow rate of 200 nl/min. The nano-LC system was hyphenated to the mass spectrometer (Q-Exactive HF, ThermoScientific, Germany) which was operated in data-dependent mode. Data interpretation was performed with Mascot V2.2 (Matrixscience, UK) and Progenesis LC–MS V4.1 (Nonlinear Dynamics, UK). Data interpretation was performed with MaxQuant V1.6.1.0 and Perseus V1.6.1.3 (MPI of Biochemistry, Germany).

### Coupling assay

Gap junction intracellular communication was tested using a dye coupling assay. Here, the fluorescent dye Calcein-AM (Sigma, 17783) was used. A neural crest population dissected from uninjected embryos was incubated with the dye Calcein-AM for approximately 10 min or until the dye was loaded into all the cells. Another neural crest population from embryos injected with a nuclear marker (nRFP mRNA injections, see above) was dissected separately. After the two populations were dissociated in absence of calcium and magnesium, they were mixed and incubated for 1 h at 14 °C in a test tube. Following mild centrifuge, the neural crest explants were cut in similar size pieces and cultured as described above and then filmed. To test the channel activity of gap junctions, gap junction blockers; meclofenamic acid (Sigma, M4531) and flufenamic acid (Sigma, F9005) were used. Cell communication was assessed by estimating the ratio of number of cells that display both tracers, the nuclear marker and the Calcein to the total number of cells that have the nuclear marker.

### Statistical analysis

The estimation of the sample size was done by following previously published work and no specific statistical method was used. Experiments were not randomized and due to the nature of the experiments, the authors were not blinded to allocation during both, experiments and results analysis. Only viable embryos and explants were analyzed. Mis-injected embryos were not included for in situ hybridization experiments. Correct injection was determined by injection of linear tracers. Our experimental parameters were measured at random once viable and properly injected embryos were selected.

Comparison of percentages was performed using contingency tables^66^. Normality of data sets was tested using Kolmogorov–Smirnov’s test, d’Agostino and Pearson’s test and Shapiro–Wilk’s test using Prism6 (GraphPad). Datasets following normal distribution were compared with Student’s *t*- test (two-tailed, unequal variances) or ANOVA with a Dunnett’s multiple comparisons post-test using Excel or Prism6 (GraphPad). Datasets that did not follow a normal distribution were compared using Mann Whitney’s test or a nonparametric ANOVA (Kruskal Wallis with Dunn’s multiple comparisons post-test) using Excel or Prism6. Cross-comparisons were performed only if the overall *p* value of the ANOVA was less than 0.05. In all figure legends *N* = number of independent experiments; *n* = total sample size.

### *X. laevis* n-cadherin partial promoter identification

To identify the basic promoter of *Xenopus* n-cad we used the following strategy. The basic promoter region from chick and mammalian n-cadherin genes have been described at the 5′-UTR regions, within the position −3000 to −1 bp respect the translation initiation site^41,67^. By using the *X. laevis* Genome Project resource (https://xenopus.lab.nig.ac.jp/) we identified a region of 2800 bp in the 5′-UTR of the *X. laevis* n-cadherin gene (Supplementary Fig. 4). Since our data indicate that Cx43Tail, BTF-3, and Pol II form a complex, we use ElemeNT tool^68^ to search for potentially active TATA boxes in the region that we isolated. Our in silico analysis reveal a TATA box rich region between the positions −166 to −618 bp, relative to the translation start site (Supplementary Fig. 4). Finally, we design overlapping primers to amplify fragments of 200 bp across this region. Primers sequences are listed in the ChIP section and their binding regions are highlighted in Supplementary Fig. 4.

### Chromatin immunoprecipitation (ChIP)

For chromatin immunoprecipitation (ChiP), we followed a standard procedure for *X. laevis* embryos^69,70^. For each independent experiment we used two technical replicas and 250–300 *Xenopus* embryos per condition. Briefly, neurula stages *X. laevis* embryos were fixed for 15 min and 3 μg of Pol II antibody (Diagenode, C15100055) or GFP ChIP grade antibody (Abcam, ab290) were used. For DNA extraction we followed a standard protocol^69,70^. Using the ElemeNT analysis resource, we searched for putative TATA boxes in the *X. laevis n-cad* promoter region (Supplementary Fig. 4). Primers flanking these TATA boxes were designed to analyze ChiP samples by PCR. PCR was performed using the following protocol 95 °C for 30 s, 56 °C for 40 s, and 72 °C for 30 s for 32 cycles. Primers used for ChiP-PCR were:

P5F: 5′-CTTCCAAGAGATGAAGCTCATAT-3′,

P5R: 5′- AACACTCTATATGGCAGATAAC-3′,

P6F: 5′-CCTTTAAATGCATACACTTACC-3′,

P6R: 5’-ACAGAAAAAGCATTTGCTTCCT-3′,

P7F: 5′-CAATCAGATCCTTATATGTCCC-3′,

P7R: 5′-GCCAAGTTTTCCCTTTGTTGT-3′,

P8F: 5′-GGAAGCAAATGCTTTTTCTGTC-3′,

P8R: 5′-AGTCTGCTTTAGGAGACAACG-3′

and their relative binding sites are shown in Supplementary Fig. 4b. ChIP experiments were quantified as follow: normalized ratio of band intensity for each condition was averaged it and the fold increase respect the IgG control was calculated and plotted as fold enrichment. Band intensity was registered by using ImageJ Gels analysis plugin. Uncropped gels are shown in Supplementary Fig. 7c, d.

## Electronic supplementary material

Supplementary Information

Description of Additional Supplementary Files

Supplementary Movie 1

Supplementary Movie 2

## Data Availability

All data and custom codes are available upon reasonable request to the corresponding author. The mass spectrometry proteomics data have been deposited to the ProteomeXchange Consortium via the PRIDE partner repository with the dataset identifier PXD010870
