# Author Correction: Gap junction protein Connexin-43 is a direct transcriptional regulator of N-cadherin in vivo

**DOI:** 10.1038/s41467-018-07167-0

**Published:** 2018-11-01

**Authors:** Maria Kotini, Elias H. Barriga, Jonathan Leslie, Marc Gentzel, Verena Rauschenberger, Alexandra Schambony, Roberto Mayor

**Affiliations:** 1 Department of Cell and Developmental Biology, University College London, Gower Street, London, WC1E 6BT UK; 2 London Centre for Nanotechnology, University College London, London, WC1H 0AH UK; 3 Max Planck Institut for Molecular Cell Biology and Genetics, Pfotenhauerstr. 108, 01307 Dresden, Germany; 4 Biology Department, Developmental Biology, Friedrich-Alexander University Erlangen-Nuremberg, Erlangen, 91058 Germany; 5 Present Address: Center for Molecular and Cellular Bioengineering, Facility Molecular Analysis/Mass Spectrometry, TU Dresden, Tatzberg 47/49, 01307 Dresden, Germany

Correction to: *Nature Communications* (2018); 10.1038/s41467-018-06368-x, published online 21 September 2018

The original version of this Article contained an error in the spelling of the author Alexandra Schambony, which was incorrectly given as Alexandra Schambon. This has now been corrected in both the PDF and HTML versions of the Article.

