# Supplementary material for: Gap junction protein Connexin-43 is a direct transcriptional regulator of N-cadherin in vivo
Source: Nat Commun. 2018 Sep 21;9:3846. doi: 10.1038/s41467-018-06368-x (PMC6155008; doi:10.1038/s41467-018-06368-x)
Supplement: Supplementary file 1 — Supplementary Information [file 41467_2018_6368_MOESM1_ESM.pdf]

Supplementary Information

Gap Junction protein Connexin-43 is a direct transcriptional regulator of N-cadherin *in vivo*

Maria Kotini, Elias H. Barriga, Jonathan Leslie, Marc Gentzl, Verena Rauschenberger, Alexandra Schambony and Roberto Mayor*

*Correspondence to: r.mayor@ucl.ac.uk

Supplementary Figures

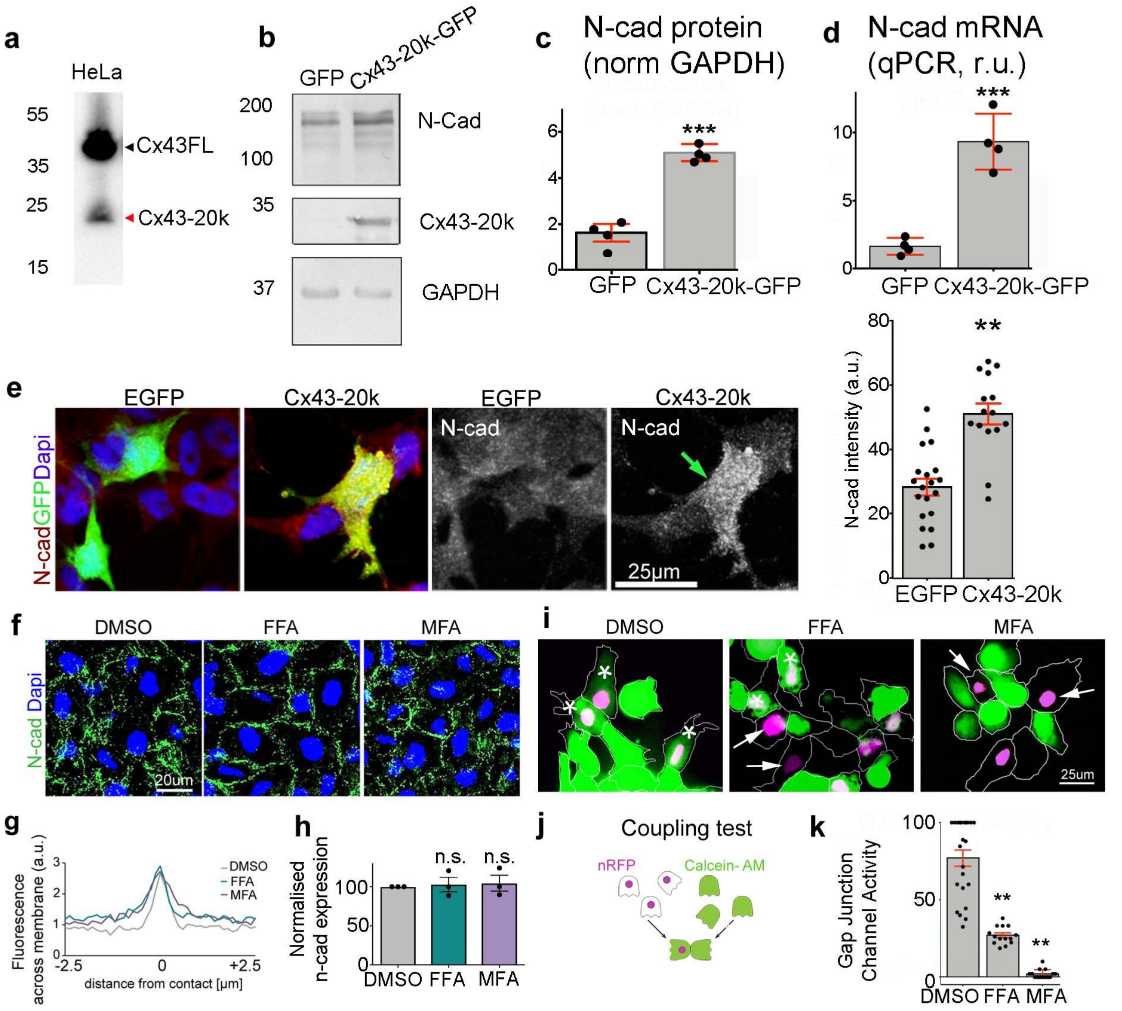

Supplementary Fig. 1. Cx43-20k upregulates N-cadherin in mammalian and amphibian cell lines. (**a-d**) HeLa cells. (**a**) Western blot with antibody against Cx43 C terminus showing the presence of Cx43FL and Cx43-20k. (**b**) Western blot with antibodies against N-cadherin, Cx43 C terminus, and GAPDH of WT HeLa cells transfected as indicated. (**c**) Quantification of N-cadherin protein from Western blot assays (N=4) (**d**) Quantification of N-cadherin mRNA by qPCR (N=4). Note that Cx43-20k increases protein and mRNA of N-cadherin in HeLa cells. (**e**) Immunostaining against N-cad on XTC cells, showing an up-regulation of N-cad in cells transfected with Cx43-20k (green arrow) (n_{EGFP}= 58, $n_{Cx43\text{-}20k}$= 69 cells, N=3). In **c**, **d** and **e** histograms represent mean ± SE, two tailed *t*-test p***<0.001, p**<0.01. In e, one-way ANOVA p<0.001. Scale bars as indicated. N= independent experiments. (**f-k**) Gap junction blockers do not affect N-cadherin levels in neural crest cells. (**f-h**) N-cadherin expression after gap junction blocker treatments. (**f**) Immunostaining for N-cad on st23 neural crests treated with DMSO or GJ blockers (FFA and MFA). (**g**) Quantification of N-cad levels across the contact, 0 represents contact point (n_{DMSO}= 35, n_{FFA}= 55, n_{MFA}= 48, N=3). (**h**) qPCR of st23 neural crest plotted as relative *n-cad* expression (n_{DMSO}= 45, n_{FFA}= 67,

n_{MFA}= 39, N=3). (**i-k**) Cell coupling after gap junction blocker treatments of st23 neural crest cells. (**i**) Coupling assay for neural crest cells cultured with DMSO, 50µM FFA or 50µM MFA. White asterisks indicate cells with GJ channel activity and arrows no channel activity. (**j**) Coupling experiment scheme. Neural crest cells were injected with mRNA encoding for nuclear RFP (nRFP) or treated with Calcein-AM. These two neural crests populations were dissociated as described in Material and Methods and mixed together. If a gap junction is formed between a Calcein-AM and a nRFP labeled cells, Calcein fluorescence should be observed in nRFP cells. (**k**) Graph showing cell communication for the various treatments. DMSO (n_{DMSO}= 28, n_{FFA}= 17, n_{MFA}= 18 explants, N=3). Note that DMSO cells display significantly higher channel activity in comparison to FFA or MFA treated cells, indicating that these blockers are efficiently impairing gap junction activity. In **h** and **k** histograms represent mean ± SE (one-way ANOVA p<0.001, two tailed *t*-test p**<0.01). Scale bars as indicated. N= number of independent experiments; n= sample size. Spread of data in bar charts is shown as overlying dots. n.s.= non-significant.

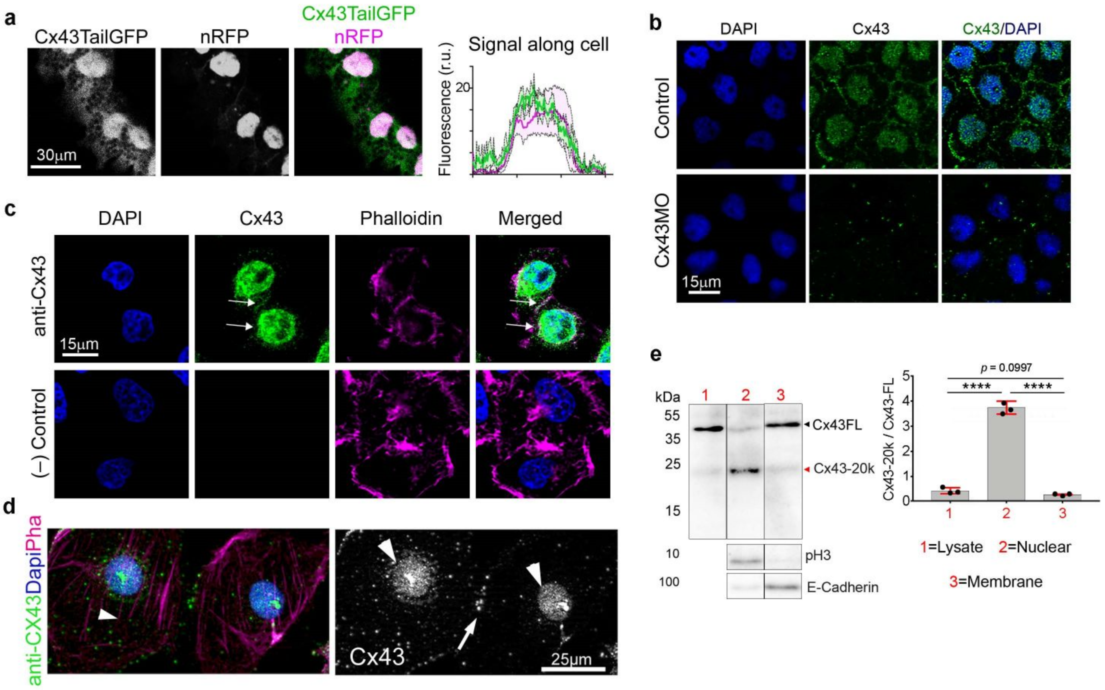

Supplementary Fig. 2. Cx43-20k is localized in the nucleus of mammalian and amphibian cells. (**a**) Neural crest cells injected with Cx43-20k-GFP. nRFP: nuclear RFP; fluorescence along cell length (N=4). Note the co-localization of GFP and RFP showing that the Cx43-20k is accumulated in the nucleus. (**b**) Immunofluorescence of st23 neural crest with antibody against the Cx43 C terminus (green). Blue: Dapi. Note localization at the cell-junction and in the nucleus. As a control neural crest were depleted of Cx43 by antisense MO (Cx43MO); note the absence of Cx43 staining. (**c**) Immunofluorescence of HeLa cells with antibody against the Cx43 C terminus (green). Blue: Dapi. Magenta: Phalloidin. Note localization of Cx43 in the nucleus and in the adhesion plaque (arrows). Control: Immunofluorescence without primary antibody against Cx43. (**d**) Immunostaining of XTC cells with antibodies against the Cx43 C terminus, which recognizes Cx43 full length and Cx43iso (N=4). Note staining at the adhesion plaque (arrow) and in the nucleus (arrowheads). Scale bars as indicated. N= independent experiments. (**e**) Western blot of nuclear and membrane fractions from *Xenopus* embryos at st23, antibodies against the Cx43 C terminus, E-cadherin (membrane marker) and p-H3 (nuclear marker) were used. Embryos were homogenized and fractionated by centrifugation. Note that Cx43-20k is enriched in the nuclear fraction confirming that the endogenous Cx43-20k is localized in the nucleus (N=3). Histograms in **e** represent mean ± S.E. (one-way ANOVA $p<0.001$, two tailed *t*-test $p^{**}<0.01$, $p^{***}<0.001$). Scale bars as indicated. N= number of independent experiments; n= sample size. Spread of data in bar charts is shown as overlying dots.

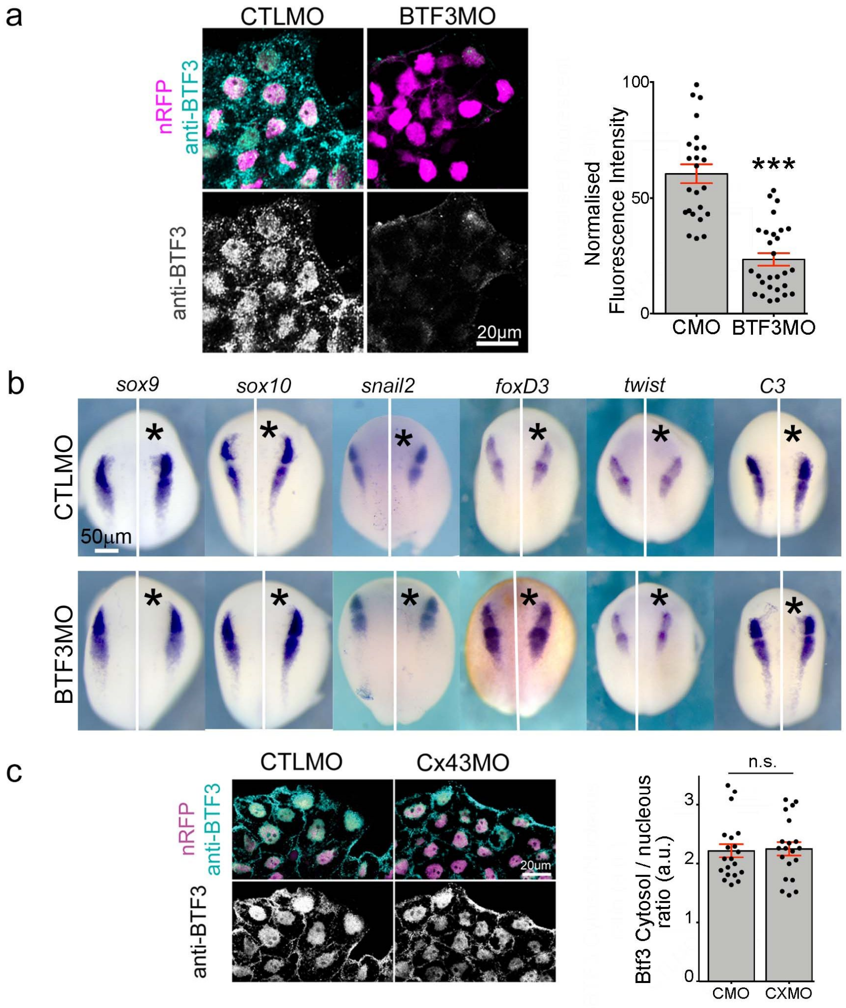

Supplementary Fig. 3. Efficiency and tissue specificity of BTF3 morpholino

(a,b) Efficiency and tissue specificity of BTF3MO *in Xenopus* neural crest. (**a**) BTF3MO leads to an efficient reduction in the endogenous levels of BTF3 protein in st23 neural crest cells. (**a**) Immunostaining for BTF3 and fluorescence levels of BTF3 normalised to background levels (n_{CMO}= 55, n_{BTF3MO}= 76, N=3). (**b**) BTF3MO does not affect neural crest induction as shown by ISH against the early neural crest markers *sox9, sox10, snail2, foxD3, twist* and *C3* at stage 16, asterisk indicates the injected side (n_{CMO}=50 embryos, n_{CxMO}=50 embryos, N=3). This result also suggests that BTF3 knock-down does not affect the expression of well-known NC transcription factors, suggesting specific interaction with Cx43-20k for the regulation of N-cadherin expression. (**c**) Nuclear localization of BTF3 in st23 neural crest is Cx43-independent. Immunostaining for BTF3 in CMO or CxMO injected neural crest and BTF3 nuclear fluorescence normalised to the cytosolic fluorescence (n_{CMO}= 22, n_{CxMO}= 43 cells, N=3). Histograms in **a** and **c** represent mean ± S.E. (two tailed *t*-test p***<0.001). Scale bars as indicated. N= number of independent experiments; n= sample size. Spread of data in bar charts is shown as overlying dots.

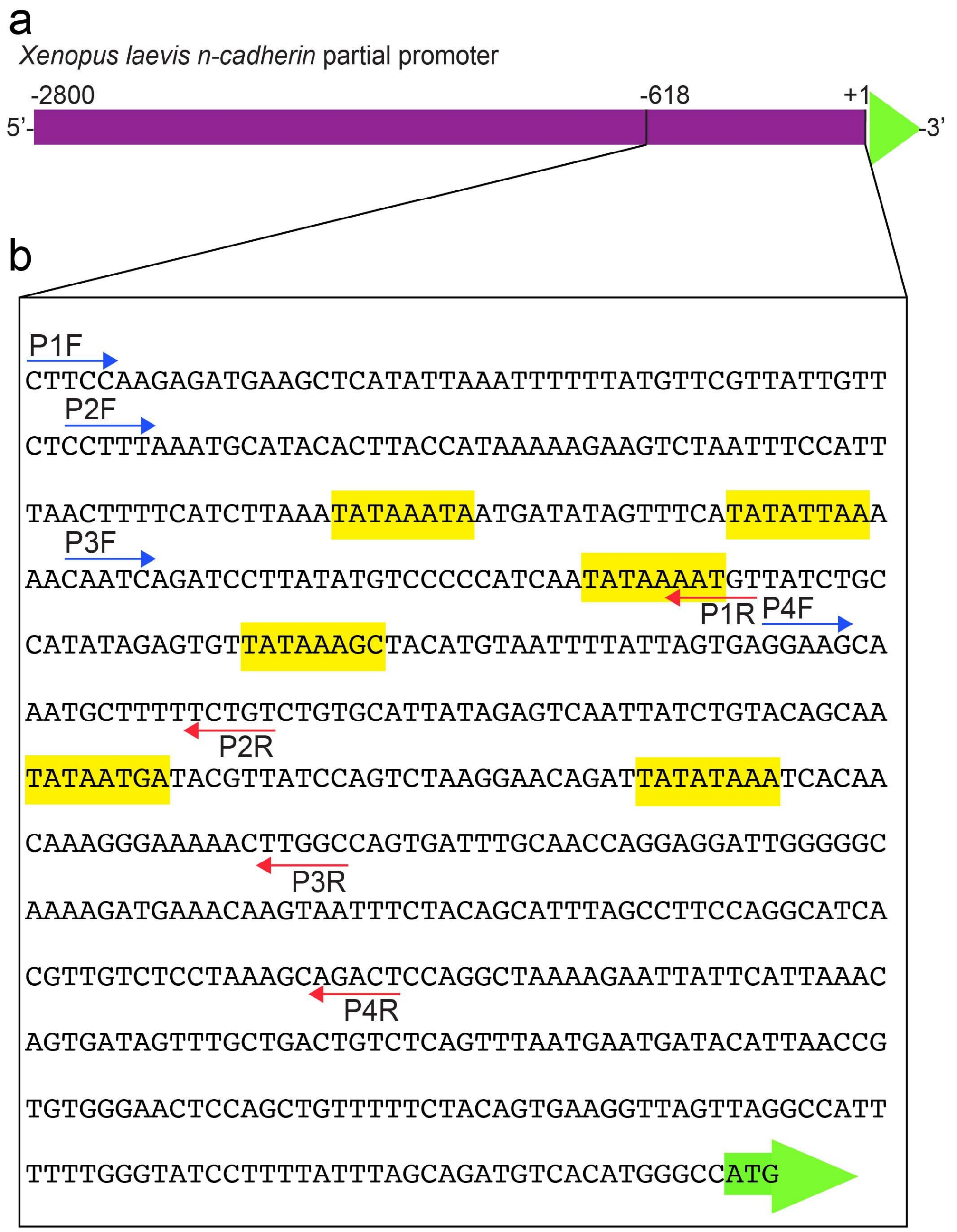

Supplementary Fig. 4. N-cadherin partial promoter from *Xenopus laevis*.
(**a**) Partial *Xenopus n-cadherin* proximal promoter region. (**b**) Putative TATA boxes on the partial promoter of *n-cad* (highlighted in yellow) and binding sites (P_nF, P_nR) for primers used in the ChIP experiments as indicated. TATA boxes obtained using ElemeNT Analysis Resource.

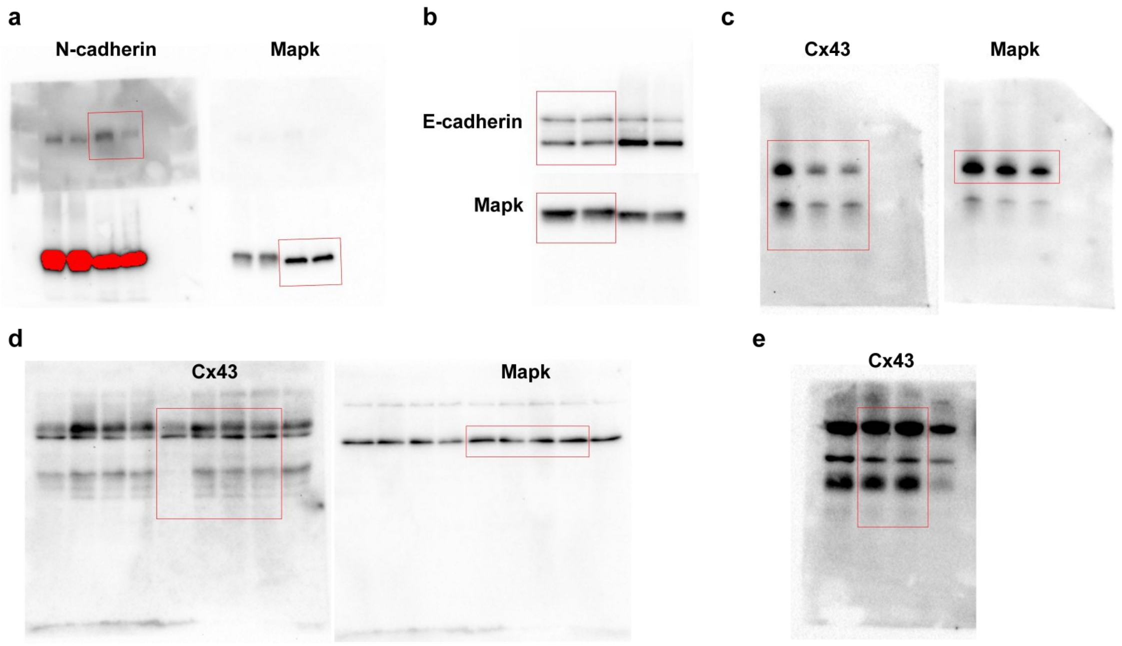

Supplementary Fig. 5. Original blot images. Uncropped blots corresponding to blot images shown in (a) Fig. 1h; (b) Fig. 1l; (c) Fig. 3a; (d) Fig. 4a; (e) Fig. 4c. Regions cropped for the main figures are highlighted in red and antibodies used for each blot are indicated in the figure.

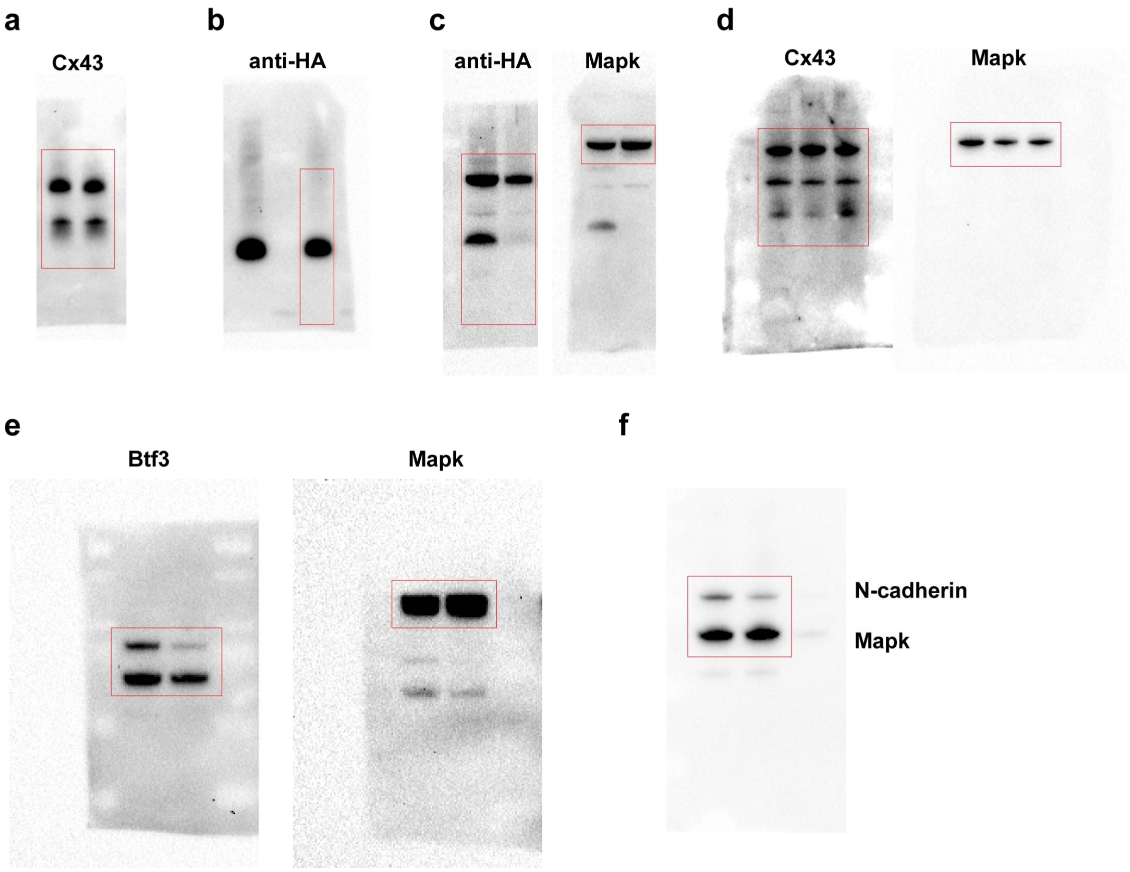

Supplementary Fig. 6. Original blot images. Uncropped blots corresponding to blot images shown in (a) Fig. 5d; (b) Fig. 5e; (c) Fig. 5g; (d) Fig. 5i; (e) Fig. 9b; (f) Fig. 9f. Regions cropped for the main figures are highlighted in red and antibodies used for each blot are indicated in the figure.

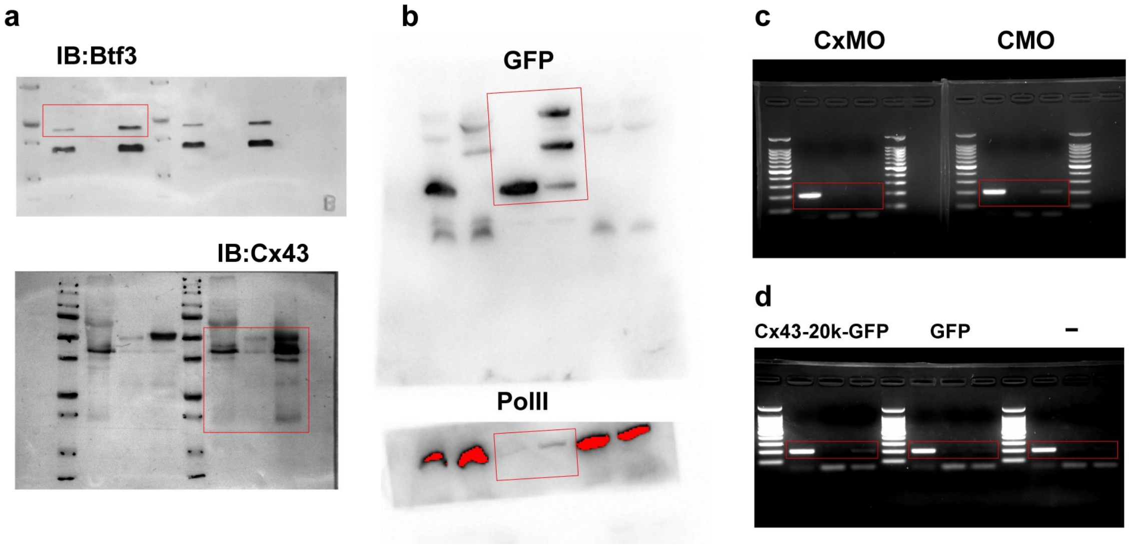

Supplementary Fig. 7. Original blot and agarose gels images. Uncropped blots corresponding to blot images shown in (a) Fig. 10d; (b) Fig. 10e; and agarose gels images shown in (c) Fig. 10f; and (d) Fig. 10g. Regions cropped for the main figures are highlighted in red and the antibodies used for each blot are indicated in the figure. Regions cropped from the agarose gels are also highlighted in red and treatments as indicated.

Supplementary Table 1. List of primers for RT-PCR

Experiment	Primer name	Sequence
qPCR	ef-1 forward	5′- ACCCTCCTCTTGGTCGTTT-3′
qPCR	ef-1 reverse	5′-TTTGGTTTTCGCTGCTTTCT-3′
qPCR	ncad forward	5′-CAGGGACCAGTTGAAGCACT-3′
qPCR	ncad reverse	5′-TGCCGTGGCCTTAAAGTTAT-3′
Chip-qPCR	P1 forward	5′-CTTCCAAGAGATGAAGCTCATAT-3′
Chip-qPCR	P1 reverse	5′- AACACTCTATATGGCAGATAAC-3′
Chip-qPCR	P2 forward	5′-CCTTTAAATGCATACACTTACC-3′
Chip-qPCR	P2 reverse	5′-ACAGAAAAAGCATTTGCTTCCT-3′
Chip-qPCR	P3 forward	5′-CAATCAGATCCTTATATGTCCC-3′
Chip-qPCR	P3 reverse	5′-GCCAAGTTTTCCCTTTGTTGT-3′
Chip-qPCR	P4 forward	5′-GGAAGCAAATGCTTTTTCTGTC-3′
Chip-qPCR	P4 reverse	5′-AGTCTGCTTTAGGAGACAACG-3′
cloning	Cx43FL forward	5′-AAAGGATCCATGGGAGATTGGAGCGCCCTCGGCAGACTT CTTGACAAAGTTCAAGC - 3′
cloning	Cx43FL reverse-tagged version	5′-AATTCTCGAGGATCTCTAAATCATCAGGTCGTG-3′
cloning	Cx43FL reverse-untagged version	5′-AATTCTCGAGTCAGATCTCCAGGTCATCAGGCC-3′
cloning	Cx43Truncated reverse	5′-AATTCTCGAGATCTTTGATACTTTTGTAGGTGAC-3′
cloning	Cx43Tail forward	5′-AAAGGATCCATGGGCATCAAAGGGAAAAAAGAC-3′
cloning	Cx43Tail-GR forward	5′-AAAGAATTCATGGGCATCAAAGGGAAAAAAGAC-3′
cloning	Cx43Tail-GR reverse	5′-AATTGAGCTCCGATCTCTAAATCATCAGGTCGTG-3′
cloning	BTF3FL forward	5′-AAAGAATTCATGAAAGAGACAATCATGAATCA-3′
cloning	BTF3FL reverse	5′-AAACTCGAGGACCGATTCATTCTTTGAAGCC-3′
cloning	BTF3-dNLS forward	5′-AAAGGATCCATGAAAGAGACAATCATGAATCA-3′
cloning	BTF3-dNLS reverse	5′-AAAATCGATTCAGACCGATTCATTCTTTGAAG-3′
cloning	BTF3-dNLS reverse tagged	5′-AAAATCGATGACCGATTCATTCTTTGAAGCC-3′
cloning	Cx43Tail for BIFC system forward	5′- AAA AAA GGG GGATCC ATGGGCATCAAAGGGAAAAAA-3′
cloning	Cx43Tail for BIFC system reverse	5′- AAA GGATCC GGAGCCGGT GATCTCTAAATCATCAGGTC-3′
cloning	BTF3 for BIFC system forward	5′- AAA AAA GGG GGATCC ATGAAAGAGACAATCATGAATCA-3′
cloning	BTF3 for BIFC system reverse	5′- AAA GGATCC GGAGGAGCCGGT GACCGATTCATTCTTTGAA-3′
cloning	Cx43 Truncated for BIFC reverse	5′- AATTGGATCCATCTTTGATACTTTTGTAGGTGAC-3′
