# Supplementary material for: Gap junction protein Connexin-43 is a direct transcriptional regulator of N-cadherin in vivo
Source: Nat Commun. 2018 Sep 21;9:3846. doi: 10.1038/s41467-018-06368-x (PMC6155008; doi:10.1038/s41467-018-06368-x)
Supplement: Supplementary file 2 — Description of Additional Supplementary Files [file 41467_2018_6368_MOESM2_ESM.pdf]

Description of Additional Supplementary Files

File Name: Supplementary Movie 1
Description: Cx43 is required for collective NC migration. Neural crest injected with control MO (left) exhibits normal chemotaxis towards a SDF-1 soaked bead; whereas in neural crest injected with a MO against Cx43 (right), chemotaxis is inhibited. Green is membrane-GFP and red/orange is nuclear-mCherry. Frame delay 10 min. Magnification 10X.

File Name: Supplementary Movie 2
Description: Cx43 is not involved in NC cell motility. Example of single cell tracks with CTLMO neural crest cells in cyan (left side) and Cx43MO in yellow (right side). Cyan and yellow are membrane-GFP and magenta is nuclear-mCherry. Frame delay 5 min. Magnification 10X.
